# Automatic detection and classification of lung cancer CT scans based on deep learning and ebola optimization search algorithm

**DOI:** 10.1371/journal.pone.0285796

**Published:** 2023-08-17

**Authors:** Tehnan I. A. Mohamed, Olaide N. Oyelade, Absalom E. Ezugwu

**Affiliations:** 1 Department of Computer Science, Faculty of Mathematical and Computer Sciences, University of Gezira, Wad Madani, Sudan; 2 School of Mathematics, Statistics, and Computer Science, University of KwaZulu-Natal, King Edward Avenue, Pietermaritzburg Campus, Pietermaritzburg, KwaZulu-Natal, South Africa; 3 Department of Computer Science, Faculty of Physical Sciences, Ahmadu Bello University, Zaria, Nigeria; 4 Unit for Data Science and Computing, North-West University, Potchefstroom, South Africa; Firat Universitesi, TURKEY

## Abstract

Recently, research has shown an increased spread of non-communicable diseases such as cancer. Lung cancer diagnosis and detection has become one of the biggest obstacles in recent years. Early lung cancer diagnosis and detection would reliably promote safety and the survival of many lives globally. The precise classification of lung cancer using medical images will help physicians select suitable therapy to reduce cancer mortality. Much work has been carried out in lung cancer detection using CNN. However, lung cancer prediction still becomes difficult due to the multifaceted designs in the CT scan. Moreover, CNN models have challenges that affect their performance, including choosing the optimal architecture, selecting suitable model parameters, and picking the best values for weights and biases. To address the problem of selecting optimal weight and bias combination required for classification of lung cancer in CT images, this study proposes a hybrid metaheuristic and CNN algorithm. We first designed a CNN architecture and then computed the solution vector of the model. The resulting solution vector was passed to the Ebola optimization search algorithm (EOSA) to select the best combination of weights and bias to train the CNN model to handle the classification problem. After thoroughly training the EOSA-CNN hybrid model, we obtained the optimal configuration, which yielded good performance. Experimentation with the publicly accessible Iraq-Oncology Teaching Hospital / National Center for Cancer Diseases (IQ-OTH/NCCD) lung cancer dataset showed that the EOSA metaheuristic algorithm yielded a classification accuracy of 0.9321. Similarly, the performance comparisons of EOSA-CNN with other methods, namely, GA-CNN, LCBO-CNN, MVO-CNN, SBO-CNN, WOA-CNN, and the classical CNN, were also computed and presented. The result showed that EOSA-CNN achieved a specificity of 0.7941, 0.97951, 0.9328, and sensitivity of 0.9038, 0.13333, and 0.9071 for normal, benign, and malignant cases, respectively. This confirms that the hybrid algorithm provides a good solution for the classification of lung cancer.

## 1. Introduction

Cancer is a severe public health issue that is becoming more prevalent worldwide. It is a disease in which cells of particular tissues undergo uncontrolled division, leading to malignant or tumor growth in the body [1]. In 2020, the GLOBOCAN estimated 19.3 million new cases of cancer and approximately 10 million cancer deaths globally [2, 3]. Lung cancer is the most commonly diagnosed cancer and the leading cause of death of men and women globally. Globally 2.2 million new lung cancer cases are diagnosed annually, which leads to close to 1.8 million deaths [2, 4]. There are several common signs and symptoms of lung cancer, including hemoptysis (coughing up blood), weight loss, and weariness. Moreover, various risk factors are associated with lung cancer, including smoking, alcohol, air quality, and food [5]. Lung cancer can be divided into two categories based on the histology of the cancer cells: small-cell lung cancer (SCLC) and non-small lung cancer (NSCLC) [1]. The NSCLC is considered the most common type of lung cancer, accounting for 85% compared to the SCLC, representing 5% of all patients [1]. Lung cancer has significantly increased in developing countries over the past two decades, including Sub-Saharan Africa, where HIV/ AIDS is also overwhelming [6]. The overall 5-year survival rate for all kinds of lung cancer is lower than 18% when compared to other cancers, such as prostate cancer (99%), colorectal cancer (65%), and breast cancer (90%) [1]. However, lung cancer demands greater attention from the medical, biological, and scientific fields to find innovative solutions to promote early diagnosis, which helps in medical decisions, and evaluates responses to improve health care. An enormous amount of computed tomography (CT) scan image data for the lungs could help detect lung cancer. Machine learning and deep learning algorithms can utilize these images to enhance cancer prediction and diagnosis as early as possible and find the best treatment strategies [7].

Deep Learning (DL) methods have enabled machines to analyze high-dimensional data such as images, multidimensional anatomical images, and videos [8, 9]. The convolutional neural network (CNN) and recurrent neural network (RNN) are popular DL models which are often applied to image and sequential data classification [10–12]. The CNN architectures are usually composed of blocks of convolutional layers and pooling operations combined with fully connected layers and a classification layer. The training process on CNN aims to tune the layers’ weights composing the architectures. This process is considered an NP-hard problem due to its susceptibility to multiple local optima requiring optimization techniques to break out of such local optima. To speed up training time and improve performance, CNNs are trained to optimize algorithms, such as stochastic gradient descent (SGD), Nesterov accelerated gradient, Adagrad, AdaDelta, and Adam, which are used to change the weights and learning rates that minimize the losses.

Building CNN architecture requires a skilful combination of hyperparameters for improved classification performance and accuracy. Approaching combinatorial problems using manual methods is daunting and reduces efficiency. However, metaheuristic algorithms have been proposed to optimize the process to obtain the best combination of hyperparameters required for improved performance. Metaheuristic algorithms are nature-inspired optimization solutions designed to help find suitable optimization constructs characterized by local search, global search and sometimes randomization and have high performances. They often require low computing capacity, which has successfully solved complex real-life problems in engineering, medical sciences, and sciences, especially in swarm intelligence algorithms [13, 14]. Considering the composition of CNN and the complexity of the hyperparameter, which requires several iterations and computational time for training its optimizers [15], the use of metaheuristic algorithms have been endorsed due to their ability to find suitable optimization constructs for overcoming limitations associated with CNN [16].

For instance, some well-known evolutionary metaheuristic algorithms are the Genetic Algorithms (GA) [17], Coral Reefs Optimization Algorithm (CRO) [18], Artificial Bee Colony (ABC) [19], Bat Algorithm (BOA) [20], Echo-cancellation Cuckoo Search Optimization (CSO) [21], Grey Wolf Optimizer (GWO) [22], Hunting Mechanism of Whale Optimization Algorithm (WOA) [23], Blue Monkey Optimization (BMO) [24], Ebola Optimization Search Algorithm (EOSA) [25, 26], Multiverse Optimizer (MVO), Whale Optimization Algorithm (WOA), Simulated Annealing (SA), Tabu Search (TS), Particle Swarm Optimization (PSO), Differential Evolution (DE), Black Hole Algorithm (BHA), Gravitational Search Algorithm (GSA), Satin Bowerbird Optimizer (SBO), Life Choice-based Optimizer (LCBO), Harmony Search (HS), and Sandpiper Optimization Algorithm (SOA) [27]. In addition, Sports-based and Light-based Algorithms are examples of Chaotic League Championship Algorithms (LCA) [28], Optics Inspired Optimization (OIO) [29], Ray Optimization (RO) [30], and Chaotic Optics-inspired Optimization (COIO) [31].

Several algorithms have been applied to medical image classification problems using CNN for feature extraction. Priyadharshini and Zoraida [32] developed Bat-inspired Metaheuristic Convolutional Neural Network Algorithms for CAD-based Lung Cancer Forecast. Li et al. [33] used metaheuristic techniques to optimize the rebalancing of the imbalanced class of feature selection method for dimension reduction in clinical X-ray image datasets. Abdullah et al. [34] applied the meta-heuristic optimization algorithm using lung images. Lu et al. [35] proposed a new convolutional neural network for the optimal detection of lung cancer. They used a marine predator metaheuristic method to improve network accuracy and optimal design. Asuntha and Srinivasan [36] presented novel deep learning methods to detect malignant lung nodules using the Fuzzy Particle Swarm Optimization (FPSO) technique to select the optimal feature after extracting texture, geometric, volumetric, and intensity information. Das et al. [37] developed a method for detecting malignant tumors by classification called Velocity-Enhanced Whale Optimization Algorithm and Artificial Neural Network to classify cancer datasets (breast, cervical, and lung cancer).

Although several studies have reported various designs of CNN algorithms developed for medical images and lung cancer prediction, there still exists some challenges due to the multifaceted designs in the CT scan. In addition, previous studies show different artichecture of the CNN model that has been used in various domains such as fabric wrinkle images [38–41]. Moreover, DL models have issues affecting their performance, including choosing the feature representation, optimal architecture, suitable model parameters, and picking the best values for weights and bias [42]. Therefore, to solve these issues of finding a precise prediction model and to advance the state-of-the-art use of CNN for the classification of lung cancer, we used metaheuristic methods to optimize the CNN model. Thus, this study proposes utilizing a metaheuristic named Ebola Optimization Search Algorithm (EOSA) [24].

The EOSA algorithm has shown promising results in various optimization problems, including feature selection and parameter optimization in different domains, such as healthcare, finance, and engineering. Furthermore, the EOSA algorithm has unique features, such as population-based search, adaptive learning, and self-learning abilities, which may have also contributed to its selection as a metaheuristic optimization method. Therefore, we selected EOSA as a metaheuristic optimization method based on its previous success in similar optimization tasks and its unique features that may provide advantages over other optimization methods. The reason for hybridizing CNN with a metaheuristic algorithm is to enhance the performance of the CNN in terms of accuracy, speed, and generalization. Metaheuristic algorithms are optimization techniques that use iterative procedures to search for the best solution in a large search space. By integrating a metaheuristic algorithm with a CNN, the model can better optimize its parameters and improve its ability to learn and classify complex patterns in the data. This can improve performance in detecting diseases like lung cancer, improving diagnostic accuracy and timely treatment. Moreover, good image preprocessing techniques, such as wavelet decomposition, will be used to enhance image resolution. As a result, this study aims to combine the EOSA-CNN algorithm with some selected image preprocessing techniques to improve the classification accuracy of the deep learning model on lung cancer CT images. The metaheuristic algorithm is applied to obtain the best combination of weights required to learn the feature extraction and classification problem.

The main objective of this article is to create an optimized deep-learning model using a metaheuristic algorithm to detect lung cancer. This model could greatly assist physicians in detecting the disease early and making informed decisions to provide suitable treatment. The following are the technical contributions of the study: (1) applied a combined wavelet decomposition and erosion, among other image preprocessing techniques, to prepare the input samples; (2) proposed a hybrid EOSA-CNN algorithm for feature extraction and classification process on the preprocessed images; and (3) evaluated and compared the hybrid algorithm with other algorithms such as GA-CNN, WOA-CNN, MVO-CN, SBO-CNN, and LCBO-CNN.

The remaining sections of the paper are organized as follows: Section 2 presents related studies on using CNN to classify lung images. In section 3, we discuss the methodology applied in this study. Section 4 presents the configuration for the experimental setup and the datasets used. Section 5 presents the results obtained and discussions on findings, while the study’s concluding remarks and future research directions are presented in Section 6.

## 2. Related works

This section reviews the application of deep learning and metaheuristics algorithms in detecting and classifying cancer cases in medical images.

Song et al. [43] developed three types of deep neural networks (CNN, DNN, and SAE) for lung cancer classification. These networks were applied to the CT image classification task with modest modifications for benign and malignant lung nodules. The CNN network showed an accuracy of 84.15%, a sensitivity of 83.96%, and a specificity of 84.32%. Bhatia et al. [44] proposed a method for detecting lung cancer from CT data using deep residual learning, which extracted features with UNet and ResNet models. The feature set was fed through multiple classifiers, including XGBoost and Random Forest, and the individual predictions were ensemble to obtain an accuracy of 84%. El-Regaily et al. [45] presented a survey of computer-aided detection systems (CAD) for lung cancer in computed tomography. They compared the current classification methods and argued that most existing algorithms could not diagnose certain forms of nodules, such as GGN. Kriegsmann et al. [46] trained and refined a CNN model to consistently classify the three most frequent lung cancer subtypes. Alrahhal and Alqhtani [47] presented ALCD, which stands for Adoptive Lung Cancer Detection, and is based on Convolutional Neural Networks (CNN). The ALCD system performed an excellent preprocessing step, and features were extracted using Scale Invariant Feature Transform, which was input into the CNN (SIFT) to perform well.

Bhandary et al. [48] provided a Deep-Learning (DL) framework for investigating lung pneumonia and cancer, which consisted of AlexNet (MAN), AlexNet, VGG16, VGG19, and ResNet50. The categorization in the MAN was done with a Support Vector Machine (SVM) and compared to Softmax. The DL framework provided an accuracy of 97.27%. Zheng et al. [49] proposed a combination of radiology analysis and malignancy evaluation network (R2MNet) to evaluate pulmonary nodule malignancy by radiology features analysis. In addition, they proposed channel-dependent activation mapping (CDAM) to visualize characteristics and shed light on the decision process of deep neural networks for model explanations (DNN) that obtained an area under the curve (AUC) of 97.52% on nodal radiology analysis. Cengil & Cinar [50] presented a classification algorithm of lung nodules using CT images of SPIE-AAPM-LungX data and a 3D CNN architecture for classification. Coudray et al. [51] trained a deep convolutional neural network (inception v3) on whole-slide images, and they yielded an average area under the curve (AUC) of 0.97. They also used the network to predict the ten most often transformed genes in LUAD. Six of them—STK11, EGFR, FAT1, SETBP1, KRAS, and TP53 were discovered to be predicted from pathology images on a held-out population, and AUCs ranged from 0.733 to 0.856. Chon et al. [52] established a CAD system for lung cancer classification of CT scans with unmarked nodules. Their initial strategy was to send segmented CT scans straight into 3D CNNs for classification, which proved insufficient.

Priyadharshini and Zoraida [32] developed Bat-inspired Metaheuristic Convolutional Neural Network Algorithms for CAD-based Lung Cancer Forecast. The Discrete Wavelet Transform (DWT) that decomposed the image as input was able to decompose the image into a set sub-band, one of which was the Low (LL) band. They used CNN to train the lung cancer data to obtain an accuracy of 97.43%. Li et al. [33] used metaheuristic techniques to optimize the rebalancing of the imbalanced class distributed to apply it in the feature selection method for dimension reduction in clinical X-ray image datasets. Using the self-adaptive Bat algorithm, feature selection with Random-SMOTE (RSMOTE) achieved 94.6% classification accuracy with 0.883 Kappa. Abdullah et al. [34] applied the meta-heuristic optimization algorithm using lung images so that features obtained were trained using convolution layers. The system’s efficiency was assessed using the F1 score value, which indicated that the system ensured a 98.9% ELT-COPD and a 98.9% NIH clinical dataset. Lu et al. [35] proposed a new convolutional neural network for the optimal detection of lung cancer using a metaheuristic method named marine predators. The proposed MPA-based approach showed 93.4% accuracy, 98.4% sensitivity, and 97.1% specificity. Asuntha and Srinivasan [36] presented a novel deep-learning method to detect malignant lung nodules and distinguish the position of the tumorous lung nodules. They used a Histogram of Oriented Gradients (HOG), wavelet transform-based features, Local Binary patterns (LBP), Scale Invariant Feature Transform (SIFT), and Zernike Moment. The Fuzzy Particle Swarm Optimization (FPSO) technique selected the optimal feature after extracting texture, geometric, volumetric, and intensity information. Das et al. [37] developed a Velocity-Enhanced Whale Optimization Algorithm, combined with an Artificial Neural Network, to classify and diagnose lung cancer. The approach is compared to C4.5, Learning Vector Quantization, Linear Discriminate Analysis, and Factorized Distribution Algorithm, giving a classification accuracy of 84%.

Senthil Kumar et al. [53] investigated and implemented new evolutionary algorithms to detect tumors and overcome the challenges related to medical image segmentation. Five evolutionary techniques were used, including k-means clustering, k-median clustering, particle swarm optimization, inertia-weighted particle swarm optimization, and guaranteed convergence particle swarm optimization (GCPSO). The GCPSO was found to have the greatest accuracy of 95.89%. Shan and Rezaei [54] designed a feature selection based on an innovative optimization method called Improved Thermal Exchange Optimization (ITEO), which aims to enhance the system’s efficiency and stability. Kapur entropy maximization and mathematical morphology were used to segment lung areas. The 19 GLCM features were collected from the segmented images for the final evaluations. ITEO used an efficient artificial neural network, and the results revealed that the proposed method attained 92.27% accuracy. Hans and Kaur [55] proposed a study that presented some of the most recent techniques. The researchers attempted to solve the lung cancer image classification challenge by utilizing some of the most recent optimization techniques. Wang et al. [56] developed a new residual neural network to determine the pathological kind of lung cancer from CT scans. They investigated a medical-to-medical transfer learning technique due to the scarcity of CT images in practice with an accuracy of 85.71%. In [57] the authors suggested a new feature selection strategy that used deep learning and integrated the Bhattacharya coefficient and genetic algorithm (GA) to pick features. Oyelade & Ezugwu [58] proposed a novel Ebola optimization search algorithm (EOSA) based on the Ebola virus and its related disease propagation model. The results showed that the proposed algorithm performed comparably to other state-of-the-art optimization approaches based on scalability, convergence, and sensitivity analyses.

Harun Bingol [59] proposed a hybrid-based deep learning model for classifying Otitis Media with Effusion (OME) based on eardrum otoendoscopic images. The proposed model combined Neighborhood Component Analysis (NCA) and the Gaussian method to extract and select features. Experimental results on a dataset comprising 910 images indicated that the proposed model achieved a high accuracy of 94.8%. Harun Bingol [59] presented a novel approach for classifying cervical cancer on Gauss-enhanced Pap-smear images using a hybrid CNN model. The performance of the proposed model was tested on a dataset comprising 1000 images, and it was found to achieve an accuracy of 93.6%, which is better than that of various other existing methods.

Therefore, considering the achievements of applying the hybrid model of CNN and optimization algorithm as reported in the studies reviewed in this section, this study aims to advance the state-of-the-art to improve lung cancer detection and classification accuracy.

## 3. Methodology

In this section, the design of the proposed hybrid EOSA-CNN algorithm is presented. A brief review of the optimization algorithm, namely the Ebola optimization search algorithm (EOSA), is presented [49]. This is followed by the design of the CNN architecture. Also, the pseudocode of the EOSA-CNN algorithm and the corresponding flowchart will be discussed in this section. The combined preprocessing techniques and the corresponding pipeline of application of the techniques are also presented.

### 3.1. The EOSA metaheuristics algorithm

We present the metaheuristic algorithm named Ebola optimization search algorithm (EOSA) based on the propagation mechanism of the Ebola virus disease [49]. The model of the EOSA algorithm is based on an improved SIR model of the disease. The model consists of the S, E, I, R, H, V, Q, and D compartments, which further translates to Susceptible (S), Exposed (E), Infected (I), Hospitalized (H), Recovered (R), Vaccinated (V), Quarantine (Q), and Death (D). The composition of these compartments allows the creation of a search space that provides optimized sets of weights and biases needed for the CNN architecture. The SIR model was then represented using a mathematical model based on a system of first-order differential equations. A combination of the propagation and mathematical models was adapted for developing the new metaheuristic algorithm. Furthermore, the resulting mathematical model was then used to design the EOSA-CNN algorithm for experimentation. The mathematical models are as follows:

$${mI}_{i}^{t+1}={mI}_{i}^{t}+\rho M(I)$$ (1)

$$\frac{\partial S(t)}{\partial t}=\pi -(\beta_{1}I+\beta_{3}D+\beta_{4}R+\beta_{2}(PE)ŋ)S-(\tau S+\Gamma I)$$ (2)

$$\frac{\partial I(t)}{\partial t}=(\beta_{1}I+\beta_{3}D+\beta_{4}R+\beta_{2}(PE)\lambda )S-(\Gamma +\gamma )I-(\tau )S$$ (3)

$$\frac{\partial H(t)}{\partial t}=\alpha I-(\gamma +\varpi )H$$ (4)

$$\frac{\partial R(t)}{\partial t}=\gamma I-\Gamma R$$ (5)

$$\frac{\partial V(t)}{\partial t}=\gamma I-(\mu +\vartheta )V$$ (6)

$$\frac{\partial D(t)}{\partial t}=(\tau S+\Gamma I)-\delta D$$ (7)

$$\frac{\partial Q(t)}{\partial t}=(\pi I-(\gamma R+\Gamma D))-\xi Q$$ (8)

In Eq (1), ρ represents the scale factor of displacement of an individual, ${mI}_{i}^{t+1}$ and ${mI}_{i}^{t}$ are the updated and original positions, respectively, at time t and t+1. Update of Susceptible (S), Infected (I), Hospitalized (H), Exposed (E), Vaccinated (V), Recovered (R), Funeral (F), Quarantine (Q), and Dead (D). A system of ordinary differential equations based on Eqs (2)–(8) are scalar functions and can be evaluated to float values. To compute these equations, the size of vectors S, I, H, R, V, D, and Q at time ***t*** are computed using initial conditions: S(0) = S_0_, I(0) = I_0_, R(0) = R_0_, D(0) = D_0_, P(0) = P_0_, and Q(0) = Q_0_ where our ***t*** follows after the definition of iterations.

The following steps describe the pseudocode of the EOSA metaheuristic algorithm:

1. Initialize all vector and scalar quantities, which are individuals and parameters: Susceptible (S), Infected (I), Recovered (R), Dead (D), Vaccinated (V), Hospitalized (H), and Quarantine (Q).
2. Randomly generate the index case (I_1_) from susceptible individuals.
3. Set the index case as the global best and current best, and compute the fitness value of the index case.
4. While the number of iterations is not exhausted and there exists at least an infected individual, then
  - Each susceptible individual generates and updates their position based on their displacement. Note that the further an infected case is displaced, the more the number of infections, so short displacement describes exploitation, otherwise exploration.
  - Generate newly infected individuals (nI) based on (a).
  - Add the newly generated cases to I.
  - Compute the number of individuals to be added to H, D, R, B, V, and Q using their respective rates based on the size of I
  - Update S and I based on new I.
  - Select the current best from I and compare it with the global best.
  - If the condition for termination is not satisfied, go back to step 4.
5. Return global best solution and all solutions.

**Fig 1:**
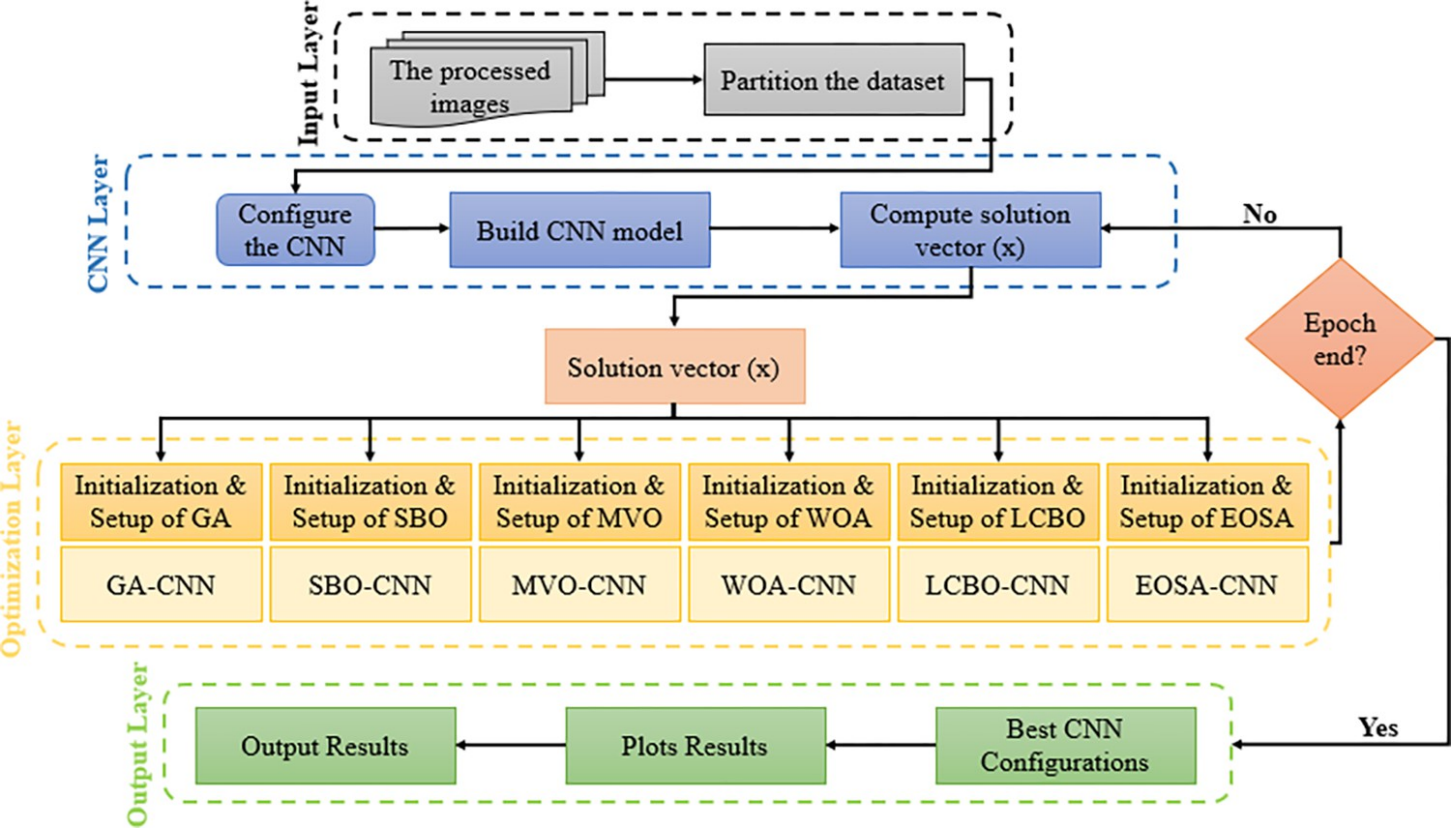
The proposed methodology.

In the following sub-sections, the application of EOSA to the optimization problem described by the study is designed and discussed. In Fig 1, an overview of the procedure for the use of the EOSA and other hybrid metaheuristic-based algorithms is presented.

### 3.2. Image preprocessing techniques

**Fig 2:**
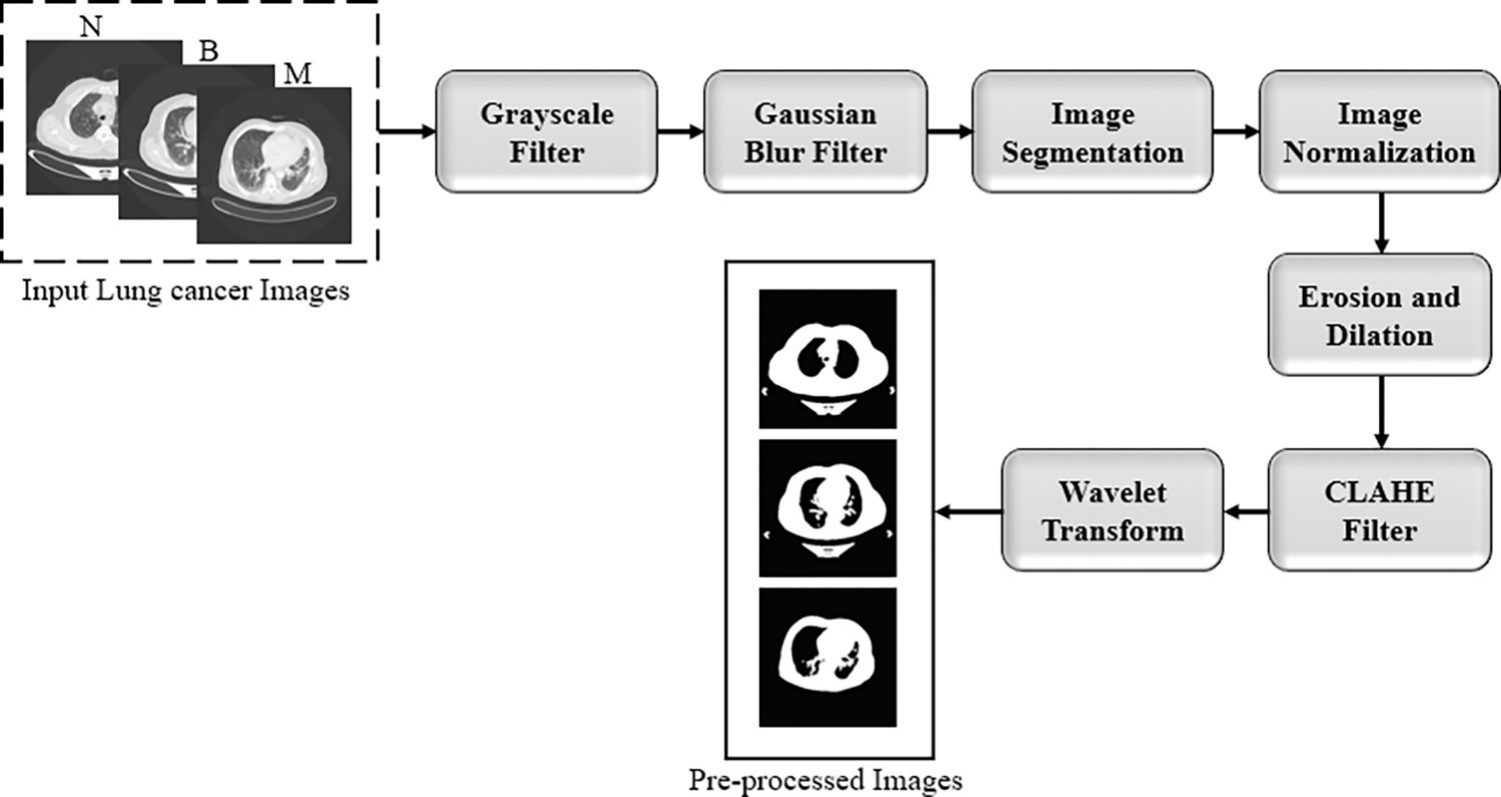
The data preprocessing steps.

Image preprocessing techniques are often applied to image samples to improve classification accuracy by removing noise and introducing sharpness [7]. The preparation of the data, also known as preprocessing, describes any processing that makes and prepares the raw data for another task, such as classification, prediction, and clustering, to ensure or enhance the task performance. In this study, the preprocessing phase includes many functions for manipulating the images into a suitable form for further analysis. Firstly, we downloaded the data from Kaggle and then read it using python. Then we applied image resizing, converting the image into the grayscale mode, Gaussian blur filter, segmentation, normalization, erosion, noise removal, and wavelet transform into the lung cancer images. Fig 2 shows the steps we followed in our preprocessing.

The Gaussian blur is a linear filter-type technique that helps image processing by implementing smoothing and blurring effects to remove the noise. It estimates the weighted mean of pixel intensities at adjacent positions. Otsu’s thresholding technique uses a threshold value that divides the image into foreground and background. The threshold value increases gradually to reach the maximum variance between the pixels of the two classes. Image normalization is an essential phase in the data preparation that changes the range of pixel intensity values. Erosion and dilation are the basic morphological operations in image processing. This process aims to extract the most relevant structure of the image viewed as a set through its subgraph representation. The mathematical equation of the erosion and dilation process is defined as shown in Eqs (9) and (10):

$$Y=A⊖B=\{x,y|{\left(B\right)}_{xy}\subseteq A\}$$ (9)

Y=A⊕B={y:B(y)∩A≠Φ} (10)

Y is a binary image, B is a template operator, and A is the original image to be processed. Image noise is the random variation of brightness or colour information in images. This noise may come from various sources, which erode image quality. We used a contrast-limited adaptive histogram equalization (CLAHE) filter to remove the unwanted noise. Wavelet analysis is a kind of multivariate analysis commonly used in medical images. The wavelet has two decomposition levels; the first level produces two coefficient vectors, namely approximation and detail coefficient, representing low and high-frequency contents. In this study, we used the biorthogonal family using *pywt*.*dwt2* function. After that, we partitioned the preprocessed data into 80% and 20% for training and testing sets, respectively. Then we built the CNN model to compute the solution vector used for the hybrid CNN-metaheuristic algorithm proposed in this study.

### 3.3. Design of the CNN architecture

**Fig 3:**
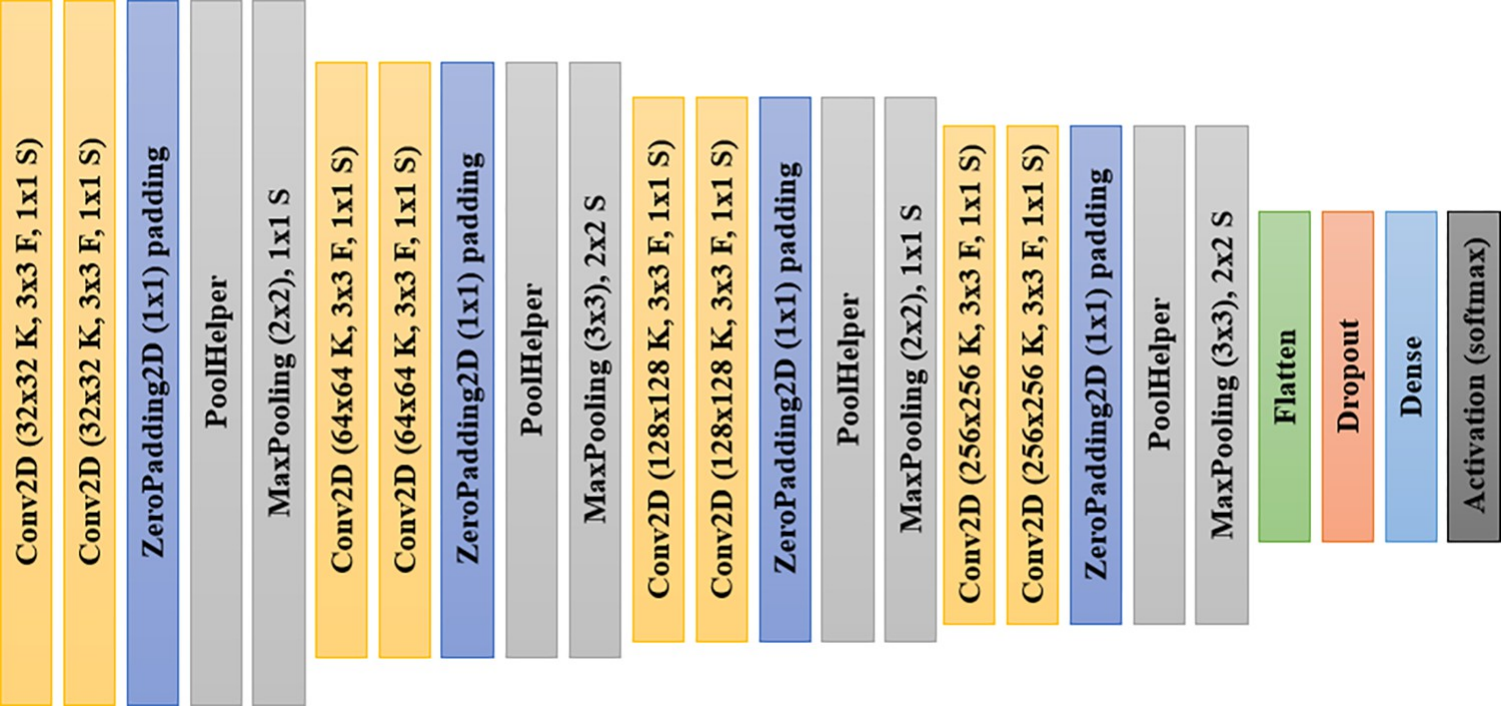
The architecture of the proposed CNN model for lung cancer detection, where F, K, and S indicate the filters, kernels, and strides, respectively.

Convolution Neural Networks (CNNs) are deep learning algorithms containing multi-layers between the input and output and are developed for image analysis and classification. Moreover, CNN is a mathematical model designed from convolution, pooling, and fully connected layers. The CNN conducts feature extraction using the convolution and pooling layers, while the fully connected layers map the extracted features into the final output. In this study, we proposed CNN architecture for design and experimentation. This architecture is depicted in Fig 3.

The CNN architecture described in Fig 3 consists of 4 blocks of convolutional-pooling layers. Each block consists of two convolutional layers, a zero-padding layer and a mas-pooling layer. The filter size and count application for the convolutional layers in the first block are 3x3 and 32x32, respectively. The PoolHelper layer is a custom layer implemented as a class and used for preselecting some features before applying the max-pooling operation. The convolutional layers in the second block consist of 64x64 filter counts and use the same 3x3 filter size as seen in the first block. The same pattern of filter size of 3x3 is seen in the convolutional blocks 3 and 4. Meanwhile, the filter count in the convolutional layers of those blocks 3 and 4 consists of 128x128 and 256x256, respectively. The max-pooling layers applied an interleaved pattern of 2x2 and 3x3 from block 1 through block 4 of the CNN architecture. After the fully connected layer appears close to the last max-pooling layer, a dropout operation uses a 0.5 drop rate. This is followed by a dense layer using the softmax function for the classification task. Feature extraction from input samples is achieved with the blocks of convolutional-pooling layers described earlier.

### 3.4. EOSA-CNN algorithm

**Fig 4:**
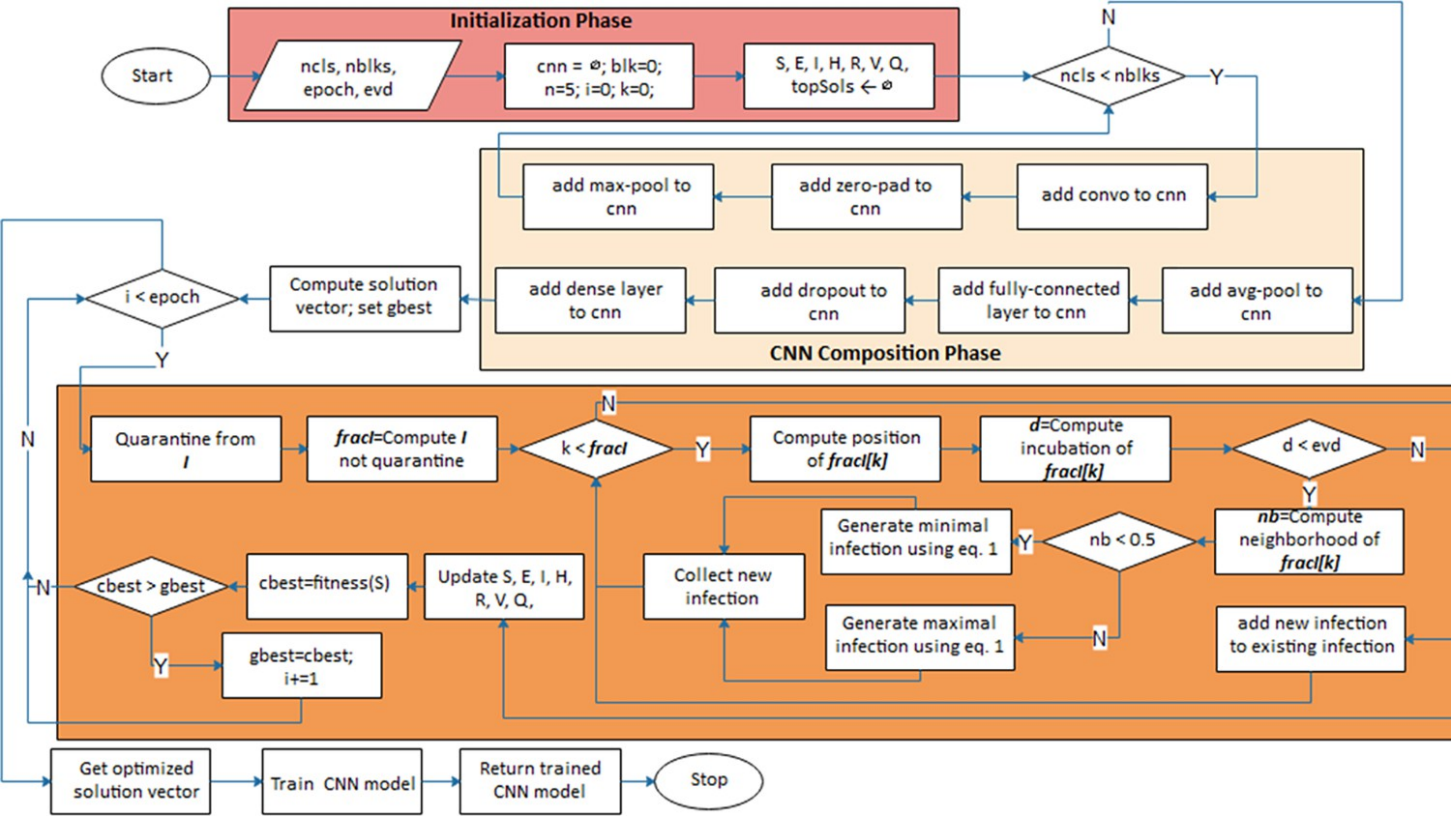
The flowchart of the EOSA-CNN algorithm showing the optimization process for the computed solution vector.

The procedure for building the proposed CNN architecture and the application of the optimization procedure is described in Fig 4. Three major phases are considered in the design: the initialization phase, the CNN composition phase, and the optimization phase. Meanwhile, we also demonstrate the need for full training in optimized CNN architecture, as seen in the flowchart. The notations *ncls*, *nblks*, *fracl*, and *evd* represent the number of convolutional layers, the number of convolutional blocks, the fraction of infected cases and the estimated virus incubation duration.

The optimization process for the CNN architecture is as follows: first, the problem size for the optimization algorithm is obtained by summing the size of the weights *w* and the bias *b* for the CNN architecture. Both *w* and *b* were obtained from the input and output sizes of the CNN, respectively. So, problem size *pz* is defined by Eq (11). Initial solutions of *pz* size were then generated, and their fitness values were computed using the Eq (12). For *t* iterations, the optimization algorithm is trained until the initial solutions improve to yield the most optimal solution for solving the classification problem. Meanwhile, for each 1,2…*t*, the fitness values of the solutions are recomputed using (12) so that the best solution is buffered. In addition, for each of those *t*, the solutions *s* are passed to the CNN architecture for reconstruction, as seen in Eq (13), and testing datasets are applied for predicting purposes. The error rate is computed and minimized further through progressive training of the optimizer to obtain an optimal solution.

pz=w+b (11)

$$fit=\frac{1}{\frac{1}{N}\sum_{n=1}^{N}\sum_{m=1}^{M}t_{nm}{log}_{2}P_{nm}+e}$$ (12)

model=cnn.setweights(s) (13)

Where *e* denotes a small value used to control the fit yielding a wrong value, note that once the best solutions are computed, the combination of weights and bias are unwound from the solutions and then plugged back into the CNN architecture for full training. The fully trained model is then applied for prediction to solve the domain problem of classifying lung cancer images.

The procedure of creating the CNN architecture, computation of the solution vector, optimization of its weights, and full training of the architecture using the optimized weights is presented in Algorithm 1. Lines 4–13 describe the configuration required to design the CNN architecture based on the parameters supplied. The solution vector of CNN architecture is then computed and supplied to the optimization algorithm as the search space in Line 20. Lines 19–22 of the algorithm show the initialization phase of the metaheuristic algorithm applied in this study.

**Algorithm 1:** EOSA-CNN algorithm

**Result:** trained CNN model

**Input:** numclasses, numblocks, kSize, epoch, psize, evdincub, objfunc

**Output:** cnn

**1**  cnn = ∅;  // initialize the model

**2** blk=0;

**3** n = 5;

**4** while *blk numblocks* do

**5** kcount=2^*n*^;

**6** cnn layer2D(kSize, kcount, relu);

**7** cnn zeropad(1);

**8 if** *blk* %*= 2* **then**

**9**  cnn maxpool(2);

**10 end**

**11 else**

**12**  cnn maxpool(3);

**13 end**

**14** n+=1;

**15 end**

**16** cnn ← avgpool(2);

**17** cnn ← flatten();

**18** cnn ← dropout(0.5); cnn dense(softmax, numclasses);

**19**  *S*, *E*, *I*, *H*, *R*, *V*, *Q*, *topSols* ← ∅

**20** *S* ← *cnn*.*getweights*()

**21** *icase* ← *S*[0]

**22** *gbest*, *cbest* ← *icase*

**23 while** *e* ≤ *epoch* ∧ *len*(*I*) *>* 0 **do**

**24** *Q* ← *rand*(0, *Eq*.8 × *I*)

**25** *fracI* = *I* − *Q*

**26 for** *i* ← 1 ***to*** *len*(*fracI*) **do**

**27**  *pos*_*i*_ ← *movrate*() *using Eq*.1

**28**  *d*_*i*_ ← *rand*()

**29  if** *d*_*i*_ *> evdincub* **then**

**30**   *neighborhood prob*(*pos*_*i*_)

**31   if** *neighborhood <* 0.5 **then**

**32**    *tmp rand*(0, *Eq*.1 *I srate*)

**33   end**

**34   else**

**35**    *tmp rand*(0, *Eq*.1 *I lrate*)

**36   end**

**37**   *newI*+ ← *tmp*

**38  end**

**39**  *I*+ ← *newI*

**40 end**

**41** *h rand*(0, *Eq*.4 *I*), *H*+ *h*

**42** *r rand*(0, *Eq*.5 *I*), *R*+ *r*

**43** *v rand*(0, *Eq*.6 *h*), *V* + *v*

**44** *d rand*(0, *Eq*.7 *I*), *D*+ *d*

**47** *S d*

**48** *cbest* = *fitness*(*objfuncs*, *S*);

**49 if** *cbest > gbest* **then**

**50**  *gbest* = *cbest*

**51**  *topSols* ← *gbest*

**52 end**

**53end**

**54** cnn.setweight(topSols)

**55** cnn.train()

**56** return cnn

Meanwhile, an index case, the infected case, is generated, and then the training process is commenced within the loop. The infected cases (s) are exposed to susceptible individuals to simulate infection, hospitalization, vaccination, dead, recovery, and quarantining in each iteration. In line 8, we showed that some infected cases (*I*) are drawn into the quarantine compartment so that only a fraction of *I* infect *S* individuals. For lines 26–40, new infections are generated from S and then added to I. Since R, V, H, and V are only derivable from I, we applied the updated *I* on Lines 41–47 to generate and update individuals using the corresponding equations. In our algorithm, recovered cases are added to S while dead individuals are replaced in S with new cases, as shown in lines 46–47. Once the loop’s termination condition is satisfied, the algorithm terminates, and the optimized solution vector is passed back to the CNN architecture for full training.

## 4. Experimentation

The experimentation to investigate the performance of the EOSA metaheuristic algorithm was first implemented, and after that, we experimented with its applicability to the hybrid EOSA-CNN algorithm. This section describes the experimental setup and parameter selection techniques used for these two experiments. Also, we present detailed datasets used in the study and demonstrate the outcome of the image preprocessing techniques applied. The benchmark functions used to evaluate the performance of the EOSA metaheuristic algorithm are also listed and discussed. Lastly, a brief discussion of evaluation metrics used to compare the performance of the hybrid’s algorithms (EOSA-CNN, GA-CNN, MVO-CNN, LCBO-CNN, WOA-CNN and SBO-CNN) are also presented.

### 4.1. Parameter settings

We conducted five experiments to independently investigate and explore the performance of the traditional CNN model and the proposed CNN using the metaheuristic optimization algorithms, including GA, SBO, MVO, WOA, LCBO, and EOSA. All the experiments were carried out on a Dell machine (Optiplex 5050) with the following specifications: Intel core i5, 7^th^ generation, 16GB memory, and 500GB hard drive. Table 1 shows the proposed CNN hyperparameter configuration.

**Table 1:** CNN hyperparameter configuration.

Parameter	Notation	CNN Architecture (C1)
Learning rate	*α*	0.0001
Loss function	*l(x)*	categorical cross entropy
Epoch	*e*	5
Batch size	*bs*	32
Optimizer	*θ*_*t*_	Adam
Kernel size/count	*f/k*	[3,3]
Convolution layers	*conv*	[2conv-2conv]
Activation function	∑*w*_*i*_*b*_*i*_	Relu
Pooling layers	*P*	[(2,2), (3,3)]
Padding/Stride	*d / s*	same / (1,1)

The input to the proposed CNN architectures is 258 × 258, representing the preprocessed images with a size of 512 × 512. Table 2 presents the metaheuristic algorithms’ configuration for optimizing the proposed CNN model. All the methods shared the same values of parameters, such as the batch size and the number of epochs.

**Table 2:** Notations and description of variables and parameters for SEIR-HDVQ.

Symbols	Descriptions	Value
π	Recruitment rate of susceptible human individuals	0.1
N	Number of iterations	100
psize	Problem size	100
R	Domain ranges (lower and upper)	[(-1, 1)]
β_1_	Contact rate of infectious human individuals	0.1
β_2_	Contact rate of pathogen individuals/environment	0.1
β_3_	Contact rate of deceased human individuals	0.1
β_4_	Contact rate of recovered human individuals	0.1
Γ	Disease-induced death rate of human individuals	[0, 1] [0, 1] range of 0–1
γ	Recovery rate of human individuals	
ŋ	Decay rate of Ebola virus in the environment	
α	Rate of hospitalization of infected individuals	
τ	Natural death rate of human individuals	
δ	Rate of burial of deceased human individuals	
ϑ	Rate of vaccination of individuals	
ϖ	Rate of response to hospital treatment	
μ	Rate of response to vaccination	
ξ	Rate of quarantine of infected individuals	

In Table 2, the initial values for each parameter are defined. Considering the stochastic nature of EOSA, which falls within the characteristic of biology-based optimization algorithms, values for some parameters are randomly assigned. The problem size applied for all experimentation is 100. We note that these values remain fixed for all experiments on the benchmark functions.

### 4.2. Datasets and image preprocessing

**Fig 5:**
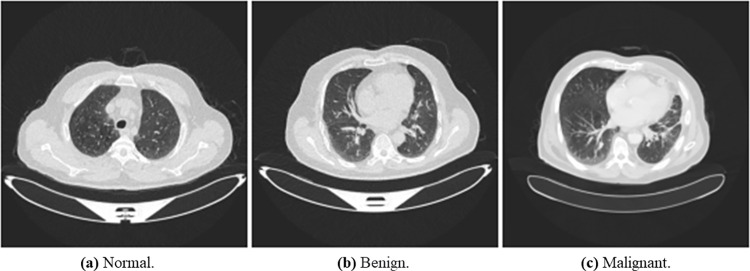
An illustration of samples from the original dataset showing images with normal, benign, and malignant labels. (a) Normal, (b) Bengin, and (c) Malignant.

We used The Iraq-Oncology Teaching Hospital/ National Center for Cancer Diseases (IQ-OTH/NCCD) lung cancer dataset (https://www.kaggle.com/kerneler/ starter-the-iq-oth-nccd-lung-cancer-09c3a8c9-4/data). This dataset was collected from two specialist hospitals for three months in 2019. The data is composed of CT scans taken from lung cancer patients diagnosed in various stages and normal patients. The data consist of 1097 samples (images) taken from 110 cases categorized into three classes: normal, benign, and malignant. One hundred and twenty (120) samples are benign, 561 samples are malignant, and 416 are normal samples. Fig 5 shows random samples of the original dataset.

**Fig 6:**
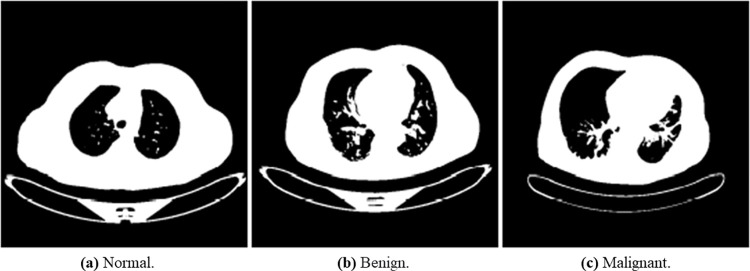
Illustration of the transformed binary images of normal, benign, and malignant samples into grayscale. (a) Normal, (b) Bengin, and (c) Malignant.

**Fig 7:**
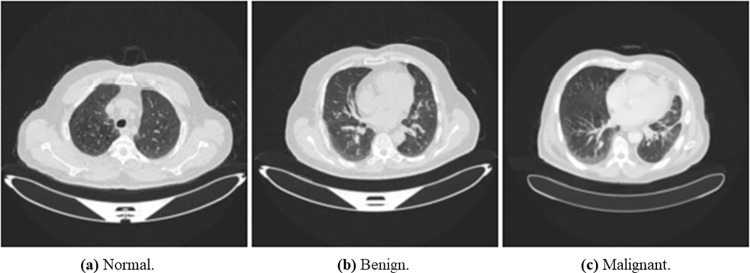
Outcome of application of Gaussian blur filter works on the lung cancer images of normal, benign, and malignant samples. (a) Normal, (b) Bengin, and (c) Malignant.

In Section 3.2, a detailed schematic diagram of the process for the image preprocessing technique applied was discussed. These techniques included grayscale, Gaussian Blur, image segmentation, image normalization, erosion and dilation CLAHE, and wavelet transform. We used the cvtColor () function in the OpenCV library to convert the lung cancer images into grayscale. The grayscale images are shown in Fig 6. We utilized the GaussianBlur () function of the OpenCV library in Python. Fig 7 below describes the results of the Gaussian blur filter on normal, benign, and malignant lung images.

**Fig 8:**
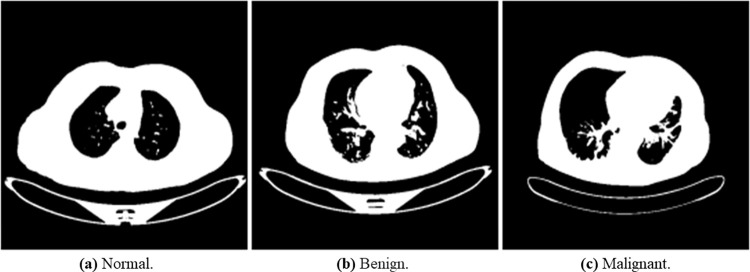
Illustrates the outcome of the Otsu’s method on normal, benign, and malignant samples. (a) Normal, (b) Bengin, and (c) Malignant.

**Fig 9:**
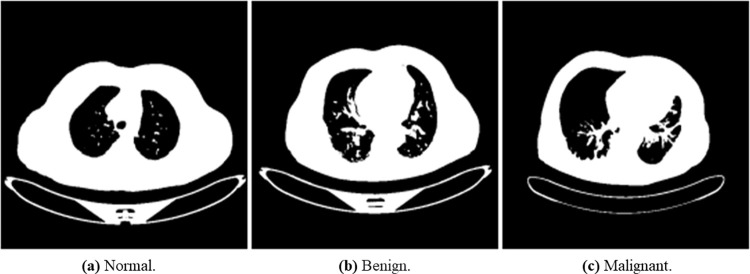
Shows normalized lung cancer on normal, benign, and malignant samples. (a) Normal, (b) Bengin, and (c) Malignant.

**Fig 10:**
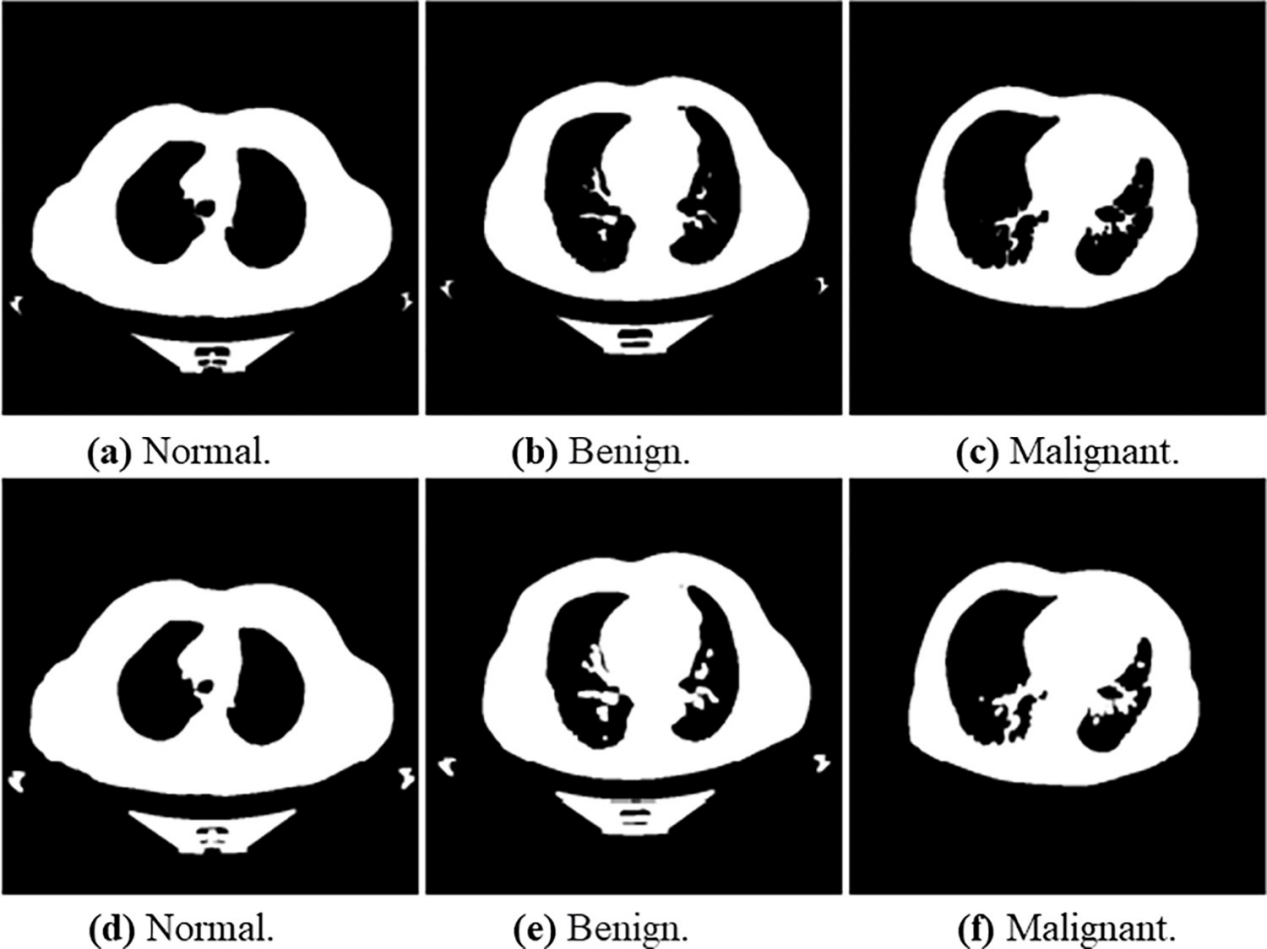
Shows the erosion (a), (b), and (c) images and dilation (d), (e), and (f) images for normal, benign, and malignant samples, respectively. (a) Normal, (b) Bengin, and (c) Malignant, (d) Normal, (e) Bengin, and (f) Malignant.

The main objective of Otsu’s method was to obtain the optimum threshold value. It can be calculated by grouping pixels into two classes, C1 and C2, and has bimodal histograms. Otsu’s method is suitable for distinguishable foreground and background with a widely reported interesting performance [60]. Considering the nature of the dataset used in this study, we applied the method for the preprocessing task. The method also reduces the intra-class variance by selecting a suitable threshold value. We used the threshold function in Python. Fig 8 below demonstrates the effects of Otsu’s method on lung cancer images. We used normalize function in Python for normalizing the lung cancer images, as seen in Fig 9. The processed lung cancer images after applying the erosion and dilation are shown in Fig 10.

**Fig 11:**
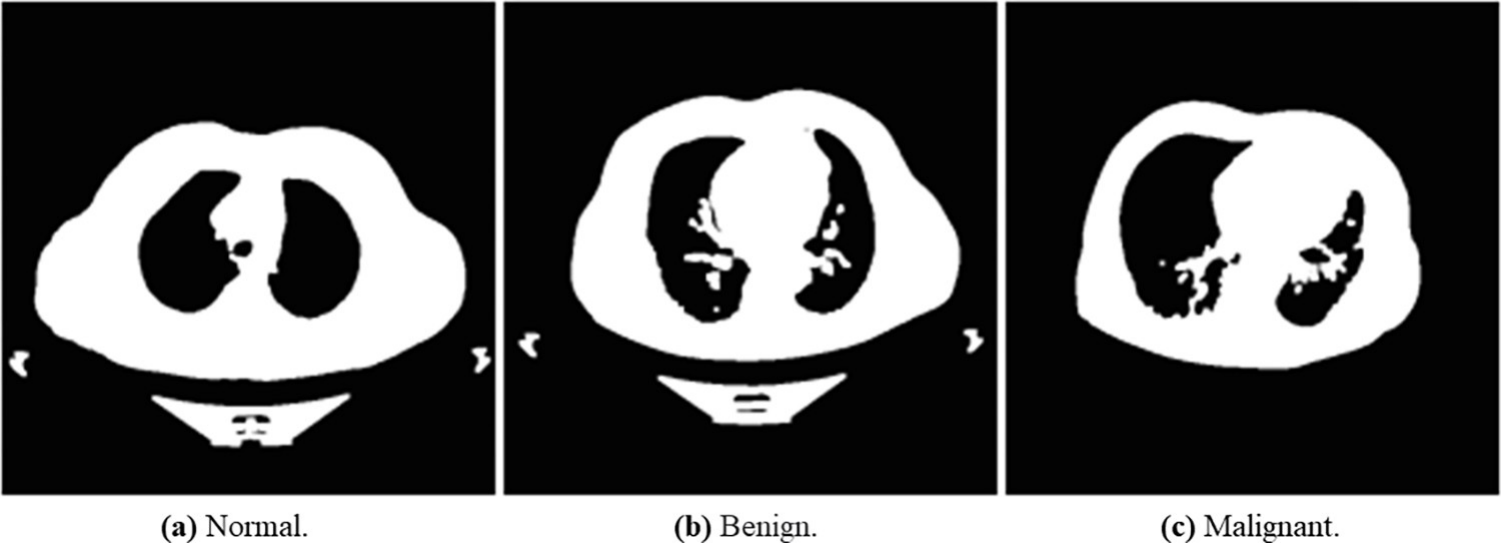
Explain the output of the CLAHE filter for normal, benign, and malignant lung cancer samples. (a) Normal, (b) Bengin, and (c) Malignant.

**Fig 12:**
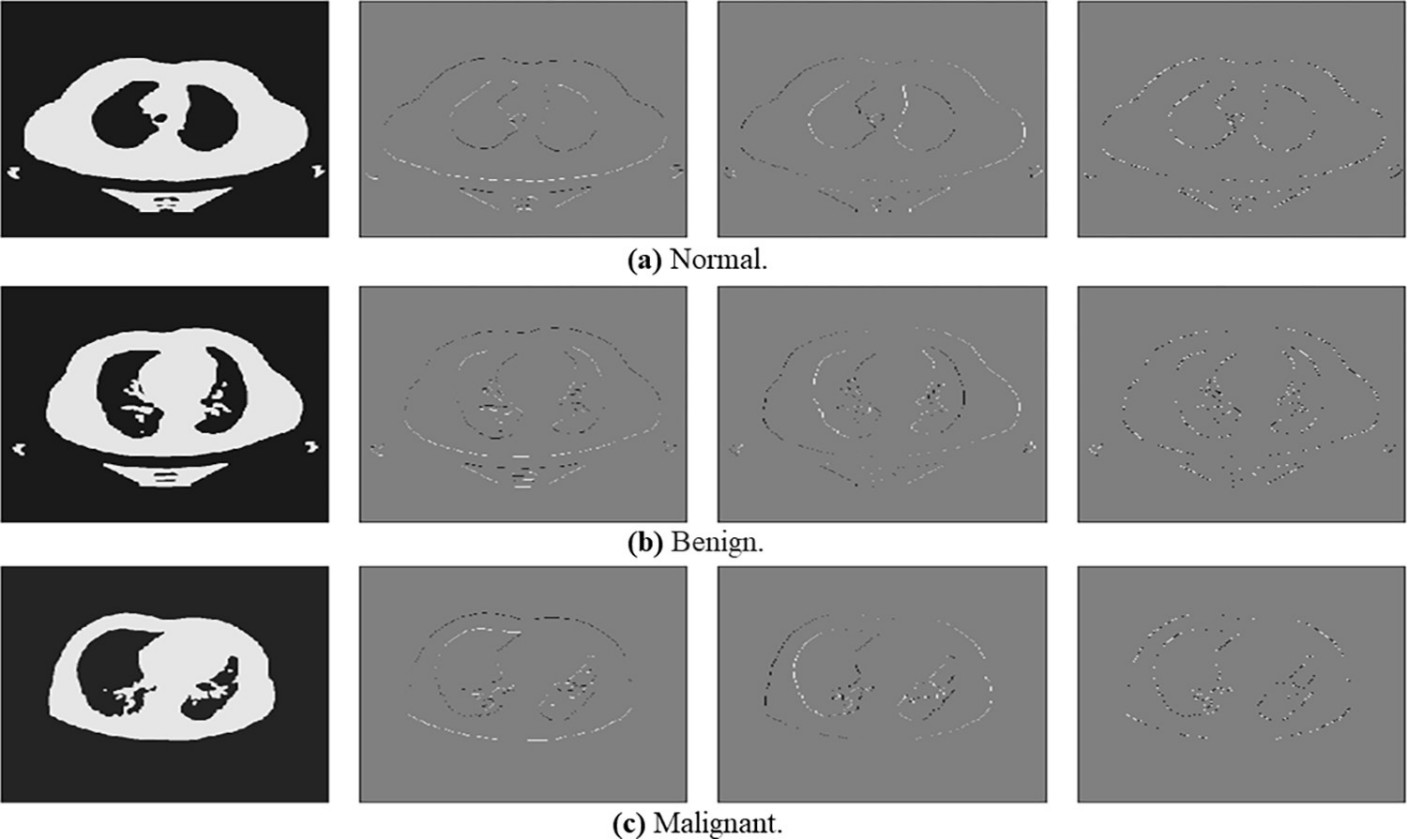
Explains the output of the wavelet filter for normal, benign, and malignant lung cancer samples. (a) Normal, (b) Bengin, and (c) Malignant.

**Fig 13:**
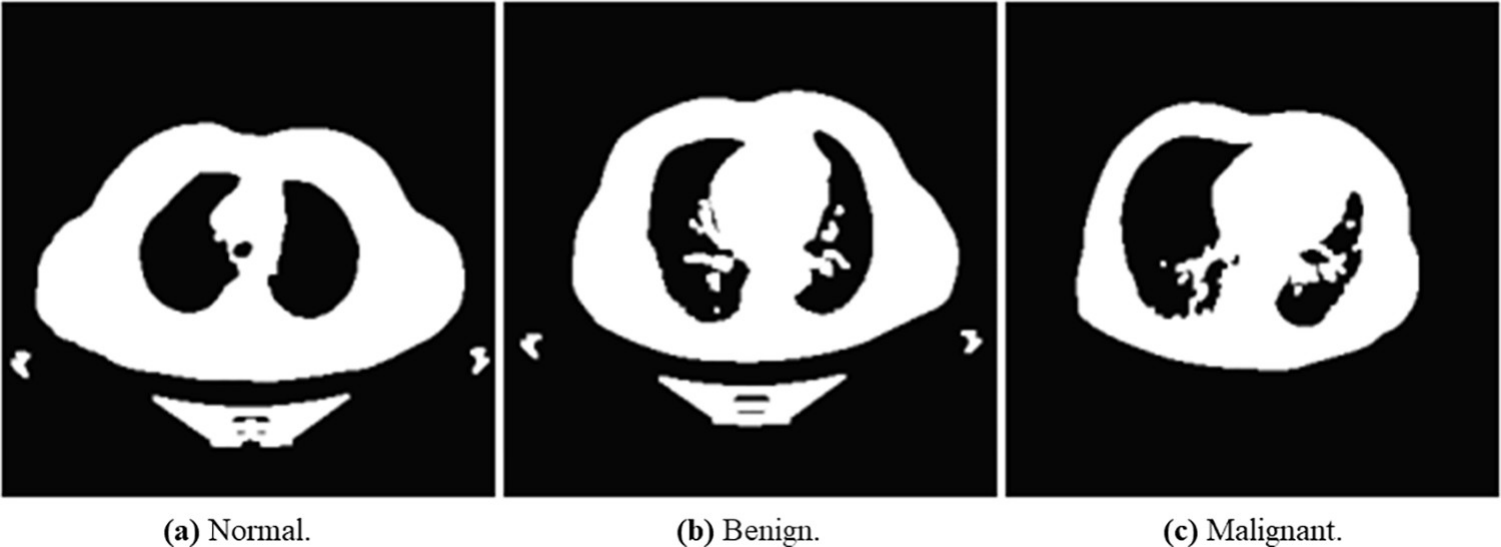
Shows the LL component of output from the wavelet filter function for normal, benign, and malignant lung cancer samples. (a) Normal, (b) Bengin, and (c) Malignant.

The result of the CLAHE filter can be seen in Fig 11. The wavelet output is depicted in Fig 12 and is decomposed into four quadrants with different interpretations (LL, LH, HL, HH). We selected the LL part for further analysis, as shown in Fig 13.

### 4.3. Benchmark functions for evaluating EOSA

To evaluate the effectiveness of the performance of the EOSA metaheuristic algorithm, we employed using 15 standard and high dimensional benchmark functions via experimentation. First, we sought to investigate the relevance of EOSA in achieving the optimization required for the classification problem. Secondly, it was necessary to compare the performance of EOSA with state-of-the-art optimization algorithms’ performance. These functions are listed in Table 3 and were subsequently used to compare similar metaheuristic algorithms in Section 5. We list the names, mathematical representations, and range values of the functions in Table 3 below.

**Table 3:** Standard benchmark functions used for the experimentation of EOSA and other similar optimization algorithms.

ID	Function name	Model of the function	Range
**F1**	Bent Cigar	$f_{20}(x)=x_{1}^{2}+10^{6}\sum_{i=2}^{D}x_{i}^{2}$	[–100,100]
**F2**	Composition2	g1 = Ackley’s Function g2 = High Conditioned Elliptic Function g3 = Griewank Function g4 = Rastrigin’s Function	[–100,100]
**F3**	Dixon and Price	$f_{18}(x)=10^{6}x_{1}^{2}\sum_{i=2}^{D}x_{i}^{2}$	[–10, 10]
**F4**	Discus Function	$f(x)={\left(x_{1}-1\right)}^{2}+\sum_{i=2}^{n}i{\left(2x_{i}^{2}-x_{i-1}\right)}^{2}$	[−100, 100]
**F5**	Fletcher–Powel	$f(x)=100\{{\left[x_{3}-10\theta (x_{1,}x_{2})\right]}^{2}+{\left(\sqrt{x_{1}^{2}+x_{2}^{2}}-1\right)}^{2}\}+x_{3}^{2}$ Where $2\pi \theta (x_{1,}x_{2})=\left\{ \begin{array}{c} {tan}^{-1}\frac{x_{2}}{x_{1}},\;if\;x_{1}\geq 0\\ \pi -{tan}^{-1}\frac{x_{2}}{x_{1}},\;otherwise \end{array}\right.$	[−100, 100]
**F6**	Generalized Penalized Function 1	$f(x)=\frac{\pi }{n}X\left\{10{sin}^{2}(\pi y_{i})+\sum_{i=1}^{n-1}{\left(y_{i}-1\right)}^{2}[1+10{sin}^{2}(\pi y_{i+1})]+{\left(y_{n}-1\right)}^{2}\right\}+\sum_{i=1}^{n}u(x_{i},a,k,m)$ Where $y_{i}=1+\frac{1}{4}(x_{i}+1),\;u(x_{i},a,k,m)=\left\{ \begin{array}{c} k{\left(x_{i}-a\right)}^{m}\;if\;x_{i}>a\\ 0\;if-a\leq x_{i}\leq a\\ k{\left(-x_{i}-a\right)}^{m}\;if\;x_{i<-a} \end{array}\right.$ a = 10, k = 100, m = 4	[–50, 50]
**F7**	Generalized Penalized Function 2	$f(x)=0.1\;X\{{sin}^{2}(3\pi x_{1})+\sum_{i=1}^{n-1}{\left(x_{i}-1\right)}^{2}[1+{sin}^{2}(3\pi x_{i+1})]+{\left(x_{n}-1\right)}^{2}[1+{sin}^{2}(2\pi x_{n})]\}+\sum_{i=1}^{n}u(x_{i},a,k,m)$ Where $u(x_{i},a,k,m)=\left\{ \begin{array}{c} k{\left(x_{i}-a\right)}^{m}\;if\;x_{i}>a\\ 0\;if-a\leq x_{i}\leq a\\ k{\left(-x_{i}-a\right)}^{m}\;if\;x_{i<-a} \end{array}\right.$ a = 5, k = 100, m = 4	[-5.12, 5.12]
**F8**	Holzman 2 function	$f(x)=\sum_{i-1}^{n}ix_{i}^{4}$	[–100,100]
**F9**	HGBat	f23(x)=|(∑i=1Dxi2)2−(∑i=1Dxi)2|12+(0.5∑i=1Dxi2+∑i=1Dxi)D+0.5	[–100,100]
**F10**	Inverted Cosine Mixture	$f_{14}(x)=0.1n-\left(0.1\sum_{i=1}^{n}cos(5\pi x_{i})-\sum_{i=1}^{n}x_{i}^{2}\right)$	[–1,1]
**F11**	Levy	$f_{12}(x)=\sum_{i=1}^{n}{\left(x_{i}-1\right)}^{2}[{sin}^{2}(3\pi x_{i+1})]+{sin}^{2}(3\pi x_{1})+|x_{n}-1|[1+{sin}^{2}(3\pi x_{n})]$	[−10, 10]
**F12**	Rosenbrock	$f(x)=\sum_{i=1}^{n-1}\left[100{\left(x_{i+1}-x_{i}^{2}\right)}^{2}+{\left(x_{i}-1\right)}^{2}\right]$	[−30, 30]
**F13**	Step	$f(x)=\sum_{i=1}^{n}{\left(floor(x_{i})+0.5\right)}^{2}$	[−100, 100]
**F14**	SR- Sum of Different Power	Shifted and Rotated Sum of Different Power Function	[–100,100]
**F15**	Wavy 1	$f(x)=\sum_{i=1}^{n}x_{i}^{2}+{\left(\sum_{i=1}^{n}0.5ix_{i}\right)}^{2}+{\left(\sum_{i=1}^{n}0.5ix_{i}\right)}^{4}$	[–100,100]

### 4.4. Classification evaluation metrics

In this paper, the comparison was based on seven performance measures, as defined in the following paragraphs. These measures were calculated from the generic confusion matrix in Table 4.

**Table 4:** Structure of the confusion matrix.

	True Condition	
**Predicted Condition**	Positive	Negative
**Positive**	True Positive (TP)	False Positive (FP)
**Negative**	False Negative (FN)	True Negative (TN)

Accuracy is the percentage of correctly classified samples:

$$Accuracy=\frac{TP+TN}{\left(TP+TN+FP+FN\right)}$$ (14)

Kappa is a chance-corrected measure of agreement between the classifications and the true classes:

$$kappa=\frac{Accuracy-Random\;Accuracy}{1-Random\;Accuracy}$$ (15)

Specificity is the proportion of actual negatives which are predicted negative:

$$Specificity=\frac{TN}{\left(TN+FP\right)}$$ (16)

Sensitivity is the proportion of actual positives which are predicted positive:

$$Sensitivity=\frac{TP}{\left(TP+FN\right)}$$ (17)

Precision is a metric which supports the ability to determine how correctly our model predicts positive cases.

$$Precision=\frac{TP}{\left(TP+FP\right)}$$ (18)

Recall is used to measure the ability of a model to pick out positive samples from the data source used for the experiment.

$$Recall=\frac{TP}{\left(TP+FN\right)}$$ (19)

F1-score is computed using a combination of recall and precision. This then allows for using the metric as the weighted average of the two underlying metrics

$$F1-score=\frac{\left(2*Precision*Recall\right)}{\left(Precision+Recall\right)}$$ (20)

## 5. Results and discussion

The performance of the proposed hybrid algorithm EOSA-CNN is evaluated in this section. The outcome of this evaluation is compared with other CNN solutions applied to the same classification problem. We also present the EOSA metaheuristic algorithms’ performance compared with other state-of-the-art methods using the benchmark functions listed in the previous section.

The performance of EOSA was compared with nine different optimization algorithms, namely Artificial Bee Colony (ABC), Whale Optimization Algorithm (WOA), Butterfly Optimization Algorithm (BOA), Particle Swarm Optimization (PSO), Differential Evolution (DE), Genetic Algorithm (GA), Henry Gas Solubility Optimization Algorithm (HGSO), Blue Monkey Optimization (BMO), and Sandpiper Optimization Algorithm (SOA). The experimentation, which was executed for five 500 iterations and 20 different runs, was applied to 15 benchmark functions.

**Fig 14:**
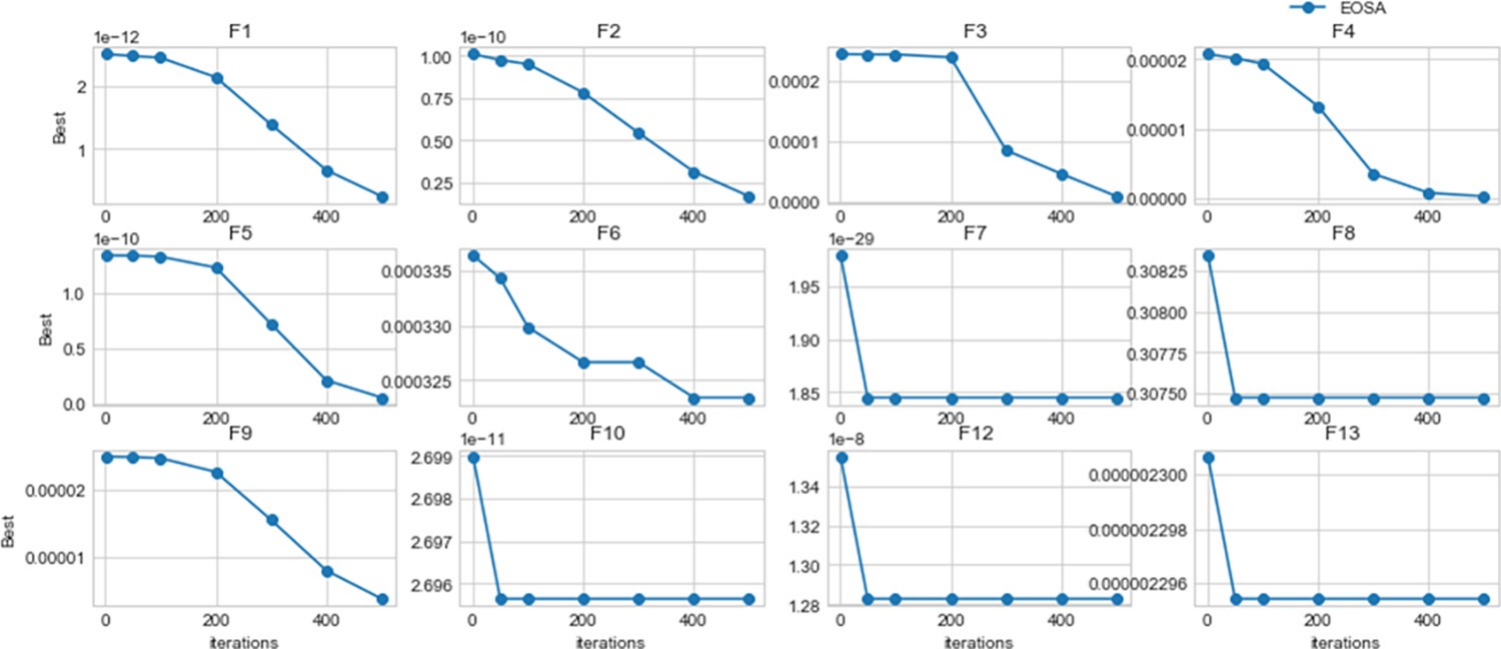
Convergent curves of EOSA on standard benchmark functions over 1, 50, 100, 200, 300, 400 and 500 epochs.

**Fig 15:**
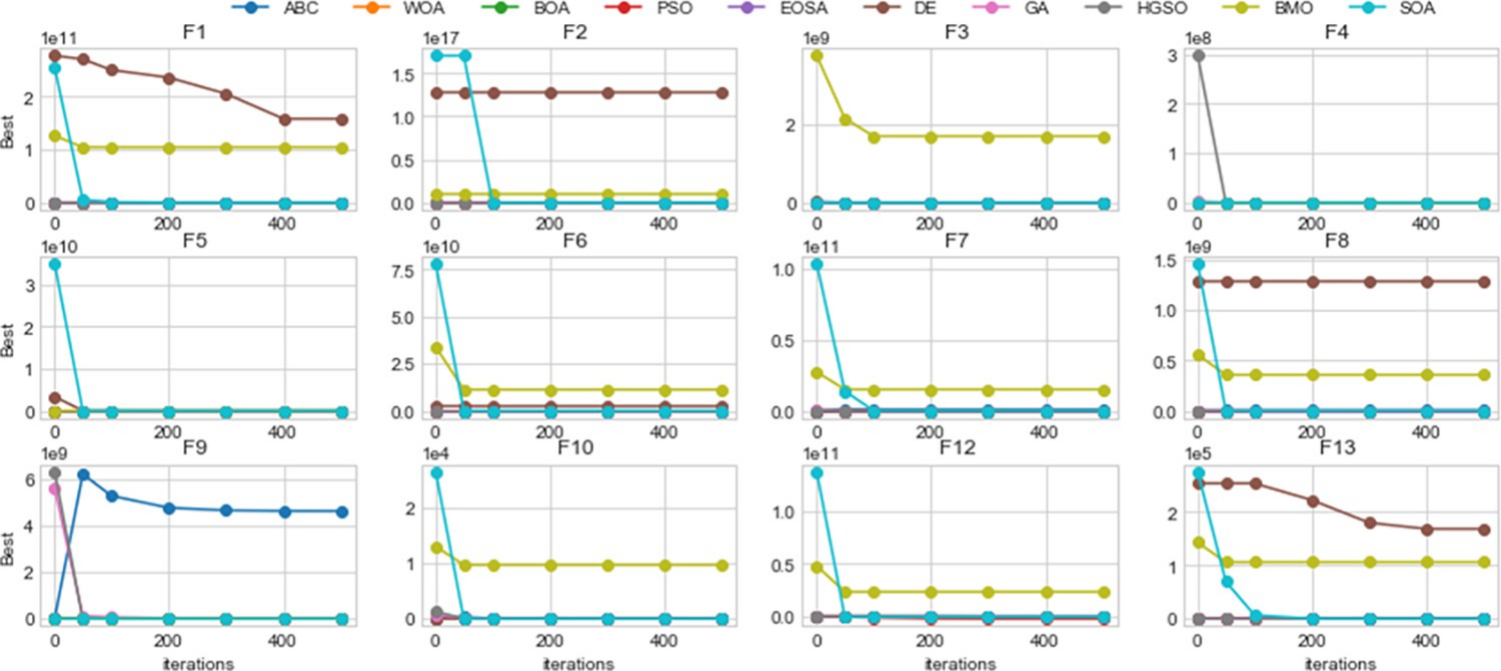
Convergent curves of EOSA and related optimization algorithms benchmark functions over 1, 50, 100, 200, 300, 400 and 500 epochs.

Using the benchmark functions listed in Table 3, the performance of EOSA compared with other state-of-the-art methods showed better outcomes, as seen in Table 5. For example, the number of times when each algorithm dominated others is described as follows: for ABC, WOA, BOA, PSO, DE, GA, BMO, EOSA, HGSO, and SOA, dominant over other methods are 2, 2, 2, 1, 1, 0, 0, 6, 1, and 4 respectively. This confirms that EOSA demonstrated superiority over other methods eight times out of all the 15 benchmark functions we experimented with. The SOA algorithm is another competitive method that follows EOSA in performance with four benchmark functions. Considering the capability of the EOSA metaheuristic algorithm to obtain more best solutions out of all the benchmark functions, it became necessary to investigate its applicability to the optimization problem described in this study. Meanwhile, Fig 14 shows a convergence graph of EOSA over some selected benchmark functions. The plot showed that the convergence pattern of the EOSA method is smooth, especially in the cases of F1-F6 and F9, and even those of F7-8 and F10-13 are seen to converge well. This demonstrates that the algorithm can search for the best solution from the global search space. This also confirms the algorithm’s applicability in solving complex real-life problems, as investigated in this study. Fig 15 shows the comparison of the convergence of EOSA with those of ABC, WOA, BOA, PSO, DE, GA, BMO, EOSA, HGSO, and SOA.

**Table 5:** Comparison of best, worst, mean, median and standard deviation values for ABC, WOA, BOA PSO, EOSA, DE, GA, HGSO, SOA, and BMO metaheuristic algorithms using the classical benchmark functions over 500 runs and 100 population size.

		ABC	WOA	BOA	PSO	DE	GA	BMO	EOSA	HGSO	SOA
**F1**	Best	2.47E-12	2.48E-12	2.50E-12	2.44E-12	1.46E+11	4106464761	1.04867E+11	2.44E-12	8.23E-104	**0**
	Worst	2.60168E+11	2.48E-12	2.50E-12	2.44E-12	2.58E+11	1.35E+11	1.26382E+11	**2.44E-12**	1.07E+11	2.55822E+11
	Mean	2.04696E+11	2.48E-12	2.50E-12	2.44E-12	2.06E+11	5647478875	1.04953E+11	**2.44E-12**	489880801.7	9790935384
**F2**	Best	2.40E-18	2.44E-18	**2.37E-18**	2.44E-18	1.49E+17	53323511.38	209785.6196	**2.37E-18**	44.10261447	44.33953792
	Worst	1.45E+17	2.44E-18	**2.37E-18**	2.44E-18	1.49E+17	2.52E+16	209785.6196	2.40E-18	1.04E+16	1.70E+17
	Mean	6.40E+16	2.44E-18	**2.37E-18**	2.44E-18	1.49E+17	5.83E+13	209785.6196	2.37E-18	3.01E+13	2.59E+16
**F3**	Best	2.86E-12	2.82E-12	2.76E-12	2.86E-12	2.71E-146	865.7595089	260316.6212	2.57E-12	1.02E-241	**0**
	Worst	100289061.5	2.82E-12	**2.76E-12**	2.86E-12	24647958.47	652837.5949	260316.6212	2.80E-12	54779492.97	5871510.079
	Mean	256702.4063	2.82E-12	2.76E-12	2.86E-12	85506.69928	26635.78996	260316.6212	**2.57E-12**	114542.9249	11743.02016
**F4**	Best	**1.01E-10**	1.02E-10	1.02E-10	**1.01E-10**	152250.3059	11996.3679	210095.2125	1.01E-10	1.22E-109	**0**
	Worst	2219235.401	1.02E-10	1.02E-10	1.01E-10	1083569.996	242291.8529	210095.2125	1.02E-10	1472206.755	482941.467
	Mean	260786.7052	1.02E-10	1.02E-10	1.01E-10	225703.8591	23799.47495	210095.2125	**1.01E-10**	5060.550052	27644.49411
**F5**	Best	1.08E-19	1.16E-19	1.13E-19	1.04E-19	**1.00E-24**	911.4244623	209785.6196	9.62E-20	0.15590465	10460.04926
	Worst	18770598227	1.16E-19	1.13E-19	1.04E-19	92699584231	1419691418	209785.6196	**9.90E-20**	3.69E+11	34961399751
	Mean	59781217.28	1.16E-19	1.13E-19	1.04E-19	238666361.4	5408441.124	209785.6196	**9.62E-20**	745438916.6	156886793.6
**F6**	Best	**1.71E-10**	1.76E-10	1.72E-10	1.73E-10	2394966487	4.159217166	11210910151	1.72E-10	1.02650854	1.187374865
	Worst	2594482104	1.76E-10	**1.72E-10**	1.73E-10	2567488472	678040718.4	33979497609	1.74E-10	429173991.9	78169609340
	Mean	975017964	1.76E-10	**1.72E-10**	1.73E-10	2524020304	1702811.703	11346936651	1.72E-10	1632585.894	5291097513
**F7**	Best	0.00537744	0.005388969	0.005480457	0.005391917	59.10789733	6.976934078	14981554900	**0.003344178**	9.850801272	9.800000008
	Worst	106.5810109	0.005388969	0.005480457	0.005391917	107.3461889	63.34963883	27264639920	**0.004373574**	43.96722626	1.03559E+11
	Mean	11.41374051	0.005388969	0.005480457	0.005391917	81.28584828	8.490634765	15100740067	**0.003346353**	10.05723641	6446484084
**F8**	Best	3.65E-10	3.74E-10	**3.66E-10**	3.73E-10	985634985.2	17956.26097	354906921.5	3.74E-10	4950	4950
	Worst	1384897512	3.74E-10	**3.66E-10**	3.73E-10	1361818654	464120523.3	554301224	3.75E-10	340835146.7	1457114306
	Mean	889078912.1	3.74E-10	**3.66E-10**	3.73E-10	1254140695	1772015.04	355869483	3.74E-10	1336939.589	24765556.99
**F9**	Best	2.45E-06	2.45E-06	2.44E-06	2.42E-06	150745.5792	4173.394244	123695.6791	**2.43E-06**	0.5	0.5
	Wworst	263287.4203	2.45E-06	**2.44E-06**	2.42E-06	262848.6996	139559.2807	140863.5674	**2.44E-06**	75064.78913	264461.4534
	Mean	205450.4186	2.45E-06	2.44E-06	2.42E-06	207583.4194	5816.112605	123809.6376	**2.43E-06**	366.1429711	7460.348419
**F10**	Best	0.065264947	0.055534741	0.065630095	0.065272135	11.29812226	4.736804364	9702.905179	0.046648475	**0**	**0**
	Worst	11.507642	0.065329978	0.065630095	0.065272135	11.44580276	10.09038934	13005.22209	**0.058911739**	8.480262936	26494.77467
	Mean	2.862967765	0.057169632	0.065630095	0.065272135	11.40128365	5.190692848	9721.634657	**0.046680024**	0.114397103	1112.80629
**F11**	Best	0.000250786	0.000246696	0.000249866	0.000252195	13.25336872	40.41662168	1985.011032	**0.000246298**	11.51810893	100
	Worst	1477.956124	0.000246696	0.000249866	0.000252195	1437.269586	820.6332779	58896.95022	**0.000247986**	669.1654077	9900
	Mean	106.6199225	0.000246696	0.000249866	0.000252195	225.3094727	57.42482498	3143.086142	**0.000246305**	29.56622817	3803.34477
**F12**	Best	4.59E-10	4.56E-10	**4.50E-10**	4.62E-10	921421360.4	16533.59328	23577837147	4.51E-10	98.86771563	98.97310275
	Worst	1085749209	4.56E-10	**4.50E-10**	4.62E-10	1105359990	365586773.6	48156845615	4.57E-10	237130959.5	1.37169E+11
	Mean	391773892	4.56E-10	**4.50E-10**	4.62E-10	1041990575	1472601.153	23631867107	4.51E-10	746936.1114	13444857087
**F13**	Best	2.47E-06	**2.43E-06**	2.45E-06	2.44E-06	149354.2611	4296.604798	105811.7963	**2.43E-06**	19.93229264	23.75105029
	Worst	257643.1306	**2.43E-06**	2.45E-06	2.44E-06	261762.2494	138652.6906	142526.1384	2.44E-06	91297.1041	275194.0507
	Mean	205778.2306	**2.43E-06**	2.45E-06	2.44E-06	204473.9907	5798.078168	106135.334	**2.43E-06**	401.736332	18094.64593
**F14**	Best	3.01E-70	3.60E-70	2.93E-70	2.61E-70	8.61E+48	1200.2428	5490765292	**2.02E-70**	1200.00029	36750667194
	Worst	4.50E+54	3.60E-70	2.93E-70	2.61E-70	7.42E+54	1.37E+51	5.41E+39	**2.53E-70**	2.72E+54	1200.052508
	Mean	1.09E+52	3.60E-70	2.93E-70	2.61E-70	5.52E+53	5.37E+48	2.73E+48	**2.02E-70**	1.08E+52	9.95E+52
**F15**	Best	2.09E-29	**1.78E-29**	1.89E-29	2.03E-29	438320.8487	110941.4238	10.88064761	1.79E-29	592240.2576	248.3259763
	Worst	2.76E+24	**1.78E-29**	1.89E-29	2.03E-29	1.10E+24	1.04E+24	1.10E+24	1.93E-29	1.92E+23	1.06326E+12
	Mean	1.43E+22	**1.78E-29**	1.89E-29	2.03E-29	3.47E+21	3.14E+21	1.10E+24	1.79E-29	6.82E+20	8.71E+18
**Total Count**		**2**	**2**	**2**	**1**	**1**	**0**	**0**	**6**	**1**	**4**

The optimized CNN architecture was fully trained to learn the classification problem of detecting and classifying lung cancer from the database samples used in the study. The trained model was then applied to a dataset for prediction. Results showed that the optimization process’s impact benefited the entire process. In Table 6, an outline of the performance of GA-CNN, LCBO-CNN, MVO-CNN, SBO-CNN, WOA-CNN, and EOSA-CNN is compared with the basic CNN with no optimization applied. The classification accuracy of EOSA-CNN yielded 0.87, demonstrating superiority over those of GA-CNN, LCBO-CNN, MVO-CNN, SBO-CNN, and WOA-CNN hybrid algorithms, which obtained 0.82, 0.83, 0.81, 0.82, 0.82, 0.87, respectively. Similarly, we observed that the EOSA-CNN algorithm demonstrated superiority over other hybrid algorithms for Kappa, recall, F1 score, and specificity by obtaining 0.70, 0.82, 0.82 and 0.98, respectively. These outcomes imply that applying the proposed EOSA-CNN hybrid algorithm benefited the classification process, leading to better classification accuracy in detecting malignancy. Furthermore, we noted that the good performance of the hybrid algorithm for specificity metric showed that it could effectively detect true negative cases, thereby reducing false-negative reports. Meanwhile, we observed that EOSA-CNN outperformed similar hybrid algorithms and outperformed the traditional CNN model, which achieved an accuracy of 0.80.

**Table 6:** The overall and per-class performance of the GA-CNN, LCBO-CNN, MVO-CNN, SBO-CNN, WOA-CNN, and EOSA-CNN hybrid algorithms as compared with the basic CNN architecture.

Measure/ Methods	GA-CNN	LCBO-CNN	MVO-CNN	SBO-CNN	WOA-CNN	EOSA-CNN	CNN
**Overall Performance**							
**Accuracy (95% CI)**	0.82	**0.83**	0.81	0.82	0.82	0.87	0.80
**Cohens kappa**	0.63	0.61	0.59	0.62	0.63	**0.70**	0.60
**Precision**	**0.84**	0.81	0.79	0.81	0.82	0.83	0.81
**Recall**	0.77	0.77	0.76	0.77	0.77	**0.82**	0.75
**F1 score**	0.80	0.78	0.77	0.79	0.79	**0.82**	0.78
**Specificity**	0.73	0.86	0.82	0.76	0.75	**0.98**	0.70
**Sensitivity**	**0.57**	0.45	0.33	0.43	0.41	0.38	0.53
**Performance per class**							
**Sensitivity**							
**Normal**	0.7212	0.8558	0.7885	0.7500	0.7404	**0.9231**	0.6827
**Benign**	0.5333	0.4333	0.3333	0.4333	0.400	0.36667	0.5333
**Malignant**	**0.8643**	0.7714	0.8214	0.8571	0.8786	0.8500	0.8500
**Specificity**							
**Normal**	**0.8941**	0.7765	0.7882	0.8235	0.8588	0.8294	0.8529
**Benign**	0.8320	0.9016	0.8893	0.8689	0.8525	**0.9508**	0.8320
**Malignant**	0.9776	0.9851	0.9701	**0.9925**	0.9851	0.9478	0.9851
**Precision**							
**Normal**	**0.8065**	0.7008	0.6949	0.7222	0.7624	0.7680	0.7396
**Benign**	0.2807	0.3514	0.2703	0.2889	0.2500	**0.4783**	0.2807
**Malignant**	0.9758	0.9818	0.9664	**0.9917**	0.984	0.9444	0.9835
**Recall**							
**Normal**	0.7212	0.8558	0.7885	0.7500	0.7404	**0.9231**	0.6827
**Benign**	**0.5333**	0.4333	0.3333	0.4333	0.400	0.3667	**0.5333**
**Malignant**	**0.8643**	0.7714	0.8214	0.8571	0.8786	0.8500	0.8500
**F1 score**							
**Normal**	0.7614	0.7706	0.7387	0.7358	0.7512	**0.8384**	0.71
**Benign**	0.36782	0.38806	0.2985	0.34667	0.3077	**0.41509**	0.36782
**Malignant**	0.9167	0.864	0.888	0.9195	**0.9283**	0.8947	0.9119
**Balanced Accuracy**							
**Normal**	0.8076	0.8161	0.7883	0.7868	0.7996	**0.8762**	0.7678
**Benign**	**0.68265**	0.66749	0.6113	0.65109	0.6262	0.65874	**0.68265**
**Malignant**	0.9209	0.8783	0.8958	0.9248	**0.9318**	0.8989	0.9175

We examined the performance of the EOSA-CNN algorithm on the three classes of labels seen on the samples drawn from the datasets. These results are listed in Table 6, where the specificity, sensitivity, precision, recall, F1-score and balanced accuracy are computed and reported. In most cases, all the hybrid algorithms competed very closely with the proposed EOSA-CNN algorithm, while it was seen to outperform the traditional CNN in most metrics. Again, this confirms that EOSA-CNN successfully indicated the features of each class and correctly classified them on an excellent performance. This further reinforces the need for the algorithm’s usefulness in addressing the classification problem in the domain.

Furthermore, a detailed report on the performance of the hybrid algorithms, when compared with the EOSA-CNN algorithm and then with the traditional CNN, is presented in Table 7, where we computed the best, mean, standard deviation, median, and worst values. These were computed for all metrics of accuracy, kappa, precision, recall, F1 score, specificity, and sensitivity for the overall performance of the algorithms. For instance, the best values obtained for accuracy for GA-CNN, LCBO-CNN, MVO-CNN, SBO-CNN, WOA-CNN, and EOSA-CNN were 0.81, 0.81, 0.79, 0.81, 0.81, and 0.82, respectively. We see that the EOSA-CNN algorithm yielded a better performance when compared with other hybrid algorithms. In addition to the EOSA-CNN surpassing other hybrids, it also outperformed the basic CNN architecture by an increase of 0.06. Similarly, the EOSA-CNN algorithm demonstrated good competitive performance with other hybrid algorithms and surpassed the traditional CNN as seen in the following: where GA-CNN, LCBO-CNN, MVO-CNN, SBO-CNN, WOA-CNN, EOSA-CNN and CNN reported for recall are 0.81, 0.81, 0.79, 0.81, 0.81, 0.82, and 0.76 respectively; GA-CNN, LCBO-CNN, MVO-CNN, SBO-CNN, WOA-CNN, EOSA-CNN and CNN reported for precision are 0.81, 0.81, 0.81, 0.82, 0.81, 0.82, and 0.78 respectively; GA-CNN, LCBO-CNN, MVO-CNN, SBO-CNN, WOA-CNN, EOSA-CNN and CNN reported for specificity are 0.9, 0.94, 0.9, 0.98, 0.94, 0.98, and 0.73 respectively.

**Table 7:** Results comparison of the best, mean, standard deviation, median, and worst overall performance based on the GA-CNN, LCBO-CNN, MVO-CNN, SBO-CNN, WOA-CNN, and EOSA-CNN hybrid algorithms compared with the basic CNN architecture.

Measure	Metric	GA-CNN	LCBO-CNN	MVO-CNN	SBO-CNN	WOA-CNN	EOSA-CNN	CNN
**Accuracy**	**Best**	0.81	0.81	0.79	0.81	0.81	**0.82**	0.76
	**Mean**	0.77	0.78	0.772	0.776	0.778	**0.81**	0.738
	**STD**	0.025495	0.018708	0.016432	0.027928	**0.031145**	0.012247	0.016432
	**Median**	0.77	0.78	0.78	0.77	0.78	**0.81**	0.73
	**Worst**	0.74	0.76	0.75	0.75	0.73	**0.79**	0.72
**Kappa**	**Best**	0.67	0.68	0.66	0.67	0.67	**0.70**	0.6
	**Mean**	0.622	0.634	0.62	0.626	0.632	**0.676**	0.574
	**STD**	0.031145	0.035071	0.03	0.042778	**0.043243**	0.018166	0.026077
	**Median**	0.62	0.64	0.63	0.62	0.64	**0.68**	0.57
	**Worst**	0.59	0.59	0.59	0.58	0.56	**0.65**	0.54
**Precision**	**Best**	0.84	**0.87**	0.85	0.86	0.83	0.83	0.81
	**Mean**	0.81	**0.824**	0.808	0.81	0.812	0.818	0.798
	**STD**	0.025495	**0.031305**	0.0249	0.030822	0.014832	0.008367	0.013038
	**Median**	0.81	0.81	0.8	0.81	0.81	**0.82**	0.8
	**Worst**	0.78	0.79	0.79	0.78	0.79	**0.81**	0.78
**Recall**	**Best**	0.81	0.81	0.79	0.81	0.81	**0.82**	0.76
	**Mean**	0.77	0.78	0.772	0.776	0.778	**0.81**	0.738
	**STD**	0.025495	0.018708	0.016432	0.027928	**0.031145**	0.012247	0.016432
	**Median**	0.77	0.78	0.78	0.77	0.78	**0.81**	0.73
	**Worst**	0.74	0.76	0.75	0.75	0.73	0.79	0.72
**F1 score**	**Best**	0.81	0.81	0.81	**0.82**	0.81	**0.82**	0.78
	**Mean**	0.786	0.79	0.782	0.788	0.79	**0.812**	0.762
	**STD**	0.018166	0.02	0.016432	**0.027749**	0.024495	0.013038	0.017889
	**Median**	0.78	0.80	0.78	0.79	0.79	**0.82**	0.76
	**Worst**	0.77	0.76	0.77	0.76	0.75	**0.79**	0.74
**Specificity**	**Best**	0.9	0.94	0.9	**0.98**	0.94	**0.98**	0.73
	**Mean**	0.782	0.828	0.83	0.824	0.808	**0.926**	0.692
	**STD**	**0.079498**	0.133866	0.072111	0.104067	0.127161	0.0498	0.023875
	**Median**	0.76	0.86	0.82	0.77	0.78	**0.92**	0.68
	**Worst**	0.7	0.62	0.72	0.73	0.64	**0.85**	0.67
**Sensitivity**	**Best**	0.66	**0.79**	0.67	0.53	0.41	0.38	0.53
	**Mean**	0.45	0.426	0.396	0.35	0.358	0.256	**0.484**
	**STD**	0.156045	**0.222778**	0.17883	0.135093	0.074297	0.11349	0.063875
	**Median**	0.37	0.37	0.37	0.33	0.37	0.27	**0.53**
	**Worst**	0.3	0.2	0.18	0.18	0.23	0.13	**0.40**

In Table 8, we compute the values for the same set of metrics, namely best, mean, standard deviation, median, and worst with respect to class labels seen in the samples from the dataset. This allows for investigating that the algorithms are not biased in detecting and classifying features from each class. We observed that results were obtained for accuracy, kappa, precision, recall, F1 score, specificity, and sensitivity for all hybrid algorithms and the traditional CNN for malignancy labels. The result obtained for the best values in all those metrics confirmed the good performance of the hybrid algorithms over the CNN architecture and for EOSA-CNN over the other hybrid algorithms. For sensitivity, GA-CNN, LCBO-CNN, MVO-CNN, SBO-CNN, and WOA-CNN obtained 0.9, 0.9214, 0.8429, 0.9071, and 0.9214, respectively, EOSA yielded 0.9071 while the CNN obtained 0.85. Similarly, for specificity, GA-CNN, LCBO-CNN, MVO-CNN, SBO-CNN, and WOA-CNN gave 0.985, 1, 1, 1, and 0.9851, respectively, but EOSA-CNN gave output 1, while the traditional CNN yielded 0.9851. These showed that for both specificity and sensitivity, the EOSA-CNN demonstrated good performance compared with the other hybrid algorithms and the basic CNN algorithm.

**Table 8:** Class-based results comparison for the best, mean, standard deviation, median, and worst of class-based performance based on the GA-CNN, LCBO-CNN, MVO-CNN, SBO-CNN, WOA-CNN, and EOSA-CNN hybrid algorithms and as compared with the basic CNN architecture.

Measure	Metric	GA-CNN	LCBO-CNN	MVO-CNN	SBO-CNN	WOA-CNN	EOSA-CNN	CNN
**Sensitivity**								
**Normal**	**Best**	0.8654	0.9368	0.875	**0.9712**	0.9135	0.9231	0.7115
	**Mean**	0.75772	0.81814	0.81156	0.8154	0.78464	**0.90**	0.67306
	**STD**	0.0791	**0.14166**	0.06252	0.10164	0.13072	0.04171	0.02451
	**Median**	0.7308	0.8558	0.8077	0.7596	0.75	**0.9231**	0.6635
	**Worst**	0.6635	0.5962	0.7212	0.7308	0.6058	**0.8269**	0.6538
**Benign**	**Best**	0.63333	**0.76667**	0.66667	0.53333	0.4	0.36667	0.53333
	**Mean**	0.42	0.41333	0.38665	0.34667	0.34666	0.24	**0.48666**
	**STD**	0.15741	**0.21551**	0.18349	0.14259	0.06912	0.10382	0.06498
	**Median**	0.36667	0.36667	0.3333	0.3333	0.36667	0.26667	**0.53333**
	**Worst**	**0.26667**	0.20	0.16667	0.16667	0.23333	0.13333	0.4
**Malignant**	**Best**	0.9	0.9214	0.8429	0.9071	**0.9214**	0.9071	0.85
	**Mean**	0.85858	0.82858	0.82286	0.83856	**0.86856**	0.86712	0.83858
	**STD**	0.03655	**0.07676**	0.01706	0.0543	0.05613	0.04213	0.01082
	**Median**	0.8643	0.8143	0.8214	0.8429	**0.8786**	0.8643	0.8357
	**Worst**	0.8143	0.7429	0.8	0.7571	0.7786	0.8071	**0.8286**
**Specificity**								
**Normal**	**Best**	0.8941	**0.9588**	0.8882	0.9059	0.8824	0.8882	0.8529
	**Mean**	**0.85296**	0.81882	0.81762	0.80234	0.83412	0.81292	0.83292
	**STD**	0.043	**0.09532**	0.04138	0.06625	0.05712	0.04893	0.01535
	**Median**	**0.8647**	0.7941	0.8059	0.7882	0.8588	0.7941	0.8353
	**Worst**	0.7824	0.7059	0.7882	0.7412	0.7588	0.7588	**0.8176**
**Benign**	**Best**	0.92623	0.93443	0.91803	0.97131	0.94262	**0.97951**	0.84836
	**Mean**	0.86803	0.8872	0.88359	0.89342	0.88443	**0.93689**	0.83361
	**STD**	0.04351	**0.05474**	0.03602	0.05011	0.05636	0.03169	0.00943
	**Median**	0.86066	0.90164	0.8893	0.877	0.877	**0.93443**	0.83197
	**Worst**	0.82377	0.80328	0.84426	0.84016	0.8115	**0.89344**	0.8238
**Malignant**	**Best**	0.985	**1**	**1**	**1**	0.9851	**1**	0.9851
	**Mean**	0.9612	0.98358	0.97462	**0.98656**	0.97016	0.96568	0.9776
	**STD**	**0.01932**	0.01435	0.01946	0.00973	0.01903	0.03192	0.0053
	**Median**	0.9552	**0.9851**	0.9701	**0.9851**	0.9776	0.9478	0.9776
	**Worst**	0.9403	0.9627	0.9478	**0.9776**	0.9403	0.9328	0.9701
**Precision**								
**Normal**	**Best**	0.8065	**0.8986**	0.8155	0.8261	0.7959	0.819	0.7396
	**Mean**	**0.76264**	0.74924	0.73322	0.72264	0.7482	0.74984	0.71148
	**STD**	0.03492	**0.09224**	0.04791	0.06092	0.03739	0.04546	0.02475
	**Median**	**0.7667**	0.7177	0.7165	0.7063	0.759	0.7328	0.7113
	**Worst**	**0.7087**	0.6599	0.6949	0.6716	0.6985	0.7007	0.6869
**Benign**	**Best**	0.30769	0.40741	0.35088	0.41667	0.42308	**0.47826**	0.30189
	**Mean**	0.28194	0.31394	0.28188	0.30389	0.29414	**0.339**	0.26439
	**STD**	0.04213	0.07393	0.06118	0.07401	**0.09156**	0.12445	0.0328
	**Median**	0.30556	0.32394	0.2703	0.29091	0.2857	**0.33333**	0.27586
	**Worst**	0.2093	0.21429	0.2	0.2105	0.1786	0.18182	**0.2182**
**Malignant**	**Best**	0.9844	**1**	**1**	**1**	0.984	**1**	0.9835
	**Mean**	0.9585	0.98232	0.97164	**0.98464**	0.96884	0.9652	0.97508
	**STD**	0.02039	0.01412	0.02146	0.01097	0.018	**0.03218**	0.00594
	**Median**	0.9508	**0.9818**	0.9672	0.9815	0.9732	0.9478	0.975
	**Worst**	0.9394	0.9627	0.9421	**0.9748**	0.9407	0.9338	0.9667
**Recall**								
**Normal**	**Best**	0.8654	0.9368	0.875	**0.9712**	0.9135	0.9231	0.7115
	**Mean**	0.75772	0.81814	0.81156	0.8154	0.78464	**0.90**	0.67306
	**STD**	0.0791	**0.14166**	0.06252	0.10164	0.13072	0.04171	0.02451
	**Median**	0.7308	0.8558	0.8077	0.7596	0.75	**0.9231**	0.6635
	**Worst**	0.6635	0.5962	0.7212	0.7308	0.6058	**0.8269**	0.6538
**Benign**	**Best**	0.63333	**0.76667**	0.66667	0.53333	0.4	0.36667	0.53333
	**Mean**	0.42	0.41333	0.38665	0.34667	0.34666	0.24	**0.48666**
	**STD**	0.15741	**0.21551**	0.18349	0.14259	0.06912	0.10382	0.06498
	**Median**	0.36667	0.36667	0.3333	0.3333	0.36667	0.26667	**0.53333**
	**Worst**	0.26667	0.2	0.16667	0.16667	0.23333	0.13333	**0.40**
**Malignant**	**Best**	0.9	**0.9214**	0.8429	0.9071	**0.9214**	0.9071	0.85
	**Mean**	0.85858	0.82858	0.82286	0.83856	**0.86856**	0.86712	0.83858
	**STD**	0.03655	**0.07676**	0.01706	0.0543	0.05613	0.04213	0.01082
	**Median**	0.8643	0.8143	0.8214	0.8429	**0.8786**	0.8643	0.8357
	**Worst**	0.8143	0.7429	0.80	0.7571	0.7786	0.8071	**0.8286**
**F1 score**								
**Normal**	**Best**	0.785	0.8128	0.8116	0.8178	0.8085	**0.8384**	0.722
	**Mean**	0.75714	0.76846	0.7691	0.76138	0.7595	**0.8164**	0.69172
	**STD**	0.02938	0.03413	0.0408	0.03759	**0.05248**	0.01586	0.02356
	**Median**	0.7614	0.7706	0.7879	0.7563	0.7723	**0.817**	0.6866
	**Worst**	0.7113	0.7168	0.7143	0.7215	0.6738	**0.7967**	0.67
**Benign**	**Best**	0.41304	0.45545	**0.45977**	0.37647	0.39286	0.41509	0.38554
	**Mean**	0.3293	0.34205	0.31888	0.30383	0.30819	0.26946	**0.34253**
	**STD**	0.06568	0.09716	0.09907	0.06419	0.06051	**0.09936**	0.04303
	**Median**	0.33333	**0.38596**	0.32099	0.3226	0.3077	0.27692	0.36364
	**Worst**	0.24658	0.23077	0.18182	0.2353	0.2326	0.15385	**0.2824**
**Malignant**	**Best**	0.9403	0.9416	0.9105	**0.9513**	0.9416	0.9272	0.9119
	**Mean**	0.90558	0.897	0.89096	0.90512	**0.91492**	0.9125	0.90168
	**STD**	0.02643	0.04082	0.01476	0.03521	0.02937	0.01712	0.00829
	**Median**	0.9118	0.8976	0.888	0.9042	**0.9236**	0.9203	0.90
	**Worst**	0.8736	0.849	0.8736	0.8548	0.8651	**0.8933**	0.8923
**Balanced Accuracy**								
**Normal**	**Best**	0.8274	0.8655	0.848	0.862	0.8508	**0.8762**	0.7764
	**Mean**	0.80532	0.81848	0.81458	0.80886	0.80936	**0.85648**	0.753
	**STD**	0.02315	0.03129	0.03384	0.03411	**0.04133**	0.01314	0.01856
	**Median**	0.8076	0.8161	0.8316	0.8033	0.8162	**0.8576**	0.7494
	**Worst**	0.77	0.7775	0.7694	0.7739	0.7441	**0.841**	0.7357
**Benign**	**Best**	0.72855	**0.78497**	0.75751	0.68675	0.6526	0.65874	0.69085
	**Mean**	0.64402	0.65027	0.63513	0.62007	0.61554	0.58844	**0.66014**
	**STD**	0.06144	**0.08653**	0.07787	0.05084	0.03404	0.04905	0.03479
	**Median**	0.6321	0.64929	0.6257	0.6216	0.6262	0.59672	**0.6806**
	**Worst**	0.58033	0.56721	0.54235	0.56899	0.5724	0.52978	**0.6119**
**Malignant**	**Best**	0.9425	0.9421	0.9179	**0.9536**	0.9421	0.9321	0.9175
	**Mean**	0.90986	0.90608	0.89874	0.91256	**0.91936**	0.91642	0.9081
	**STD**	0.02425	**0.03313**	0.01405	0.0302	0.02448	0.0146	0.00747
	**Median**	0.913	0.9071	0.8958	0.9102	**0.9237**	0.92	0.9067
	**Worst**	0.881	0.8677	0.881	0.8711	0.8781	0.8989	**0.8994**

Furthermore, we observed that for precision, GA-CNN, LCBO-CNN, MVO-CNN, SBO-CNN, WOA-CNN and EOSA-CNN obtained 0.9844, 1, 1, 1, 0.984, and 1, while CNN gave 0.9835; for recall, GA-CNN, LCBO-CNN, MVO-CNN, SBO-CNN, WOA-CNN and EOSA-CNN yielded 0.9, 0.9214, 0.8429, 0.9071, 0.9214, and 0.9071 respectively, while the traditional CNN obtained 0.85. In both cases of recall and precision, EOSA-CNN and the other hybrid CNN algorithms performed well. Also, we see that for F1 scores, 0.9403, 0.9416, 0.9105, 0.9513, 0.9416, 0.9272, and 0.9119 were reported for GA-CNN, LCBO-CNN, MVO-CNN, SBO-CNN, WOA-CNN, EOSA-CNN and CNN, while 0.9425, 0.9421, 0.9179, 0.9536, 0.9421, 0.9321 and 0.9175 were obtained for GA-CNN, LCBO-CNN, MVO-CNN, SBO-CNN, WOA-CNN, EOSA-CNN and CNN respectively with respect to balanced accuracy. A good competitive performance is seen for the classification accuracy of all hybrid algorithms, with the basic CNN architecture lagging.

**Fig 16:**
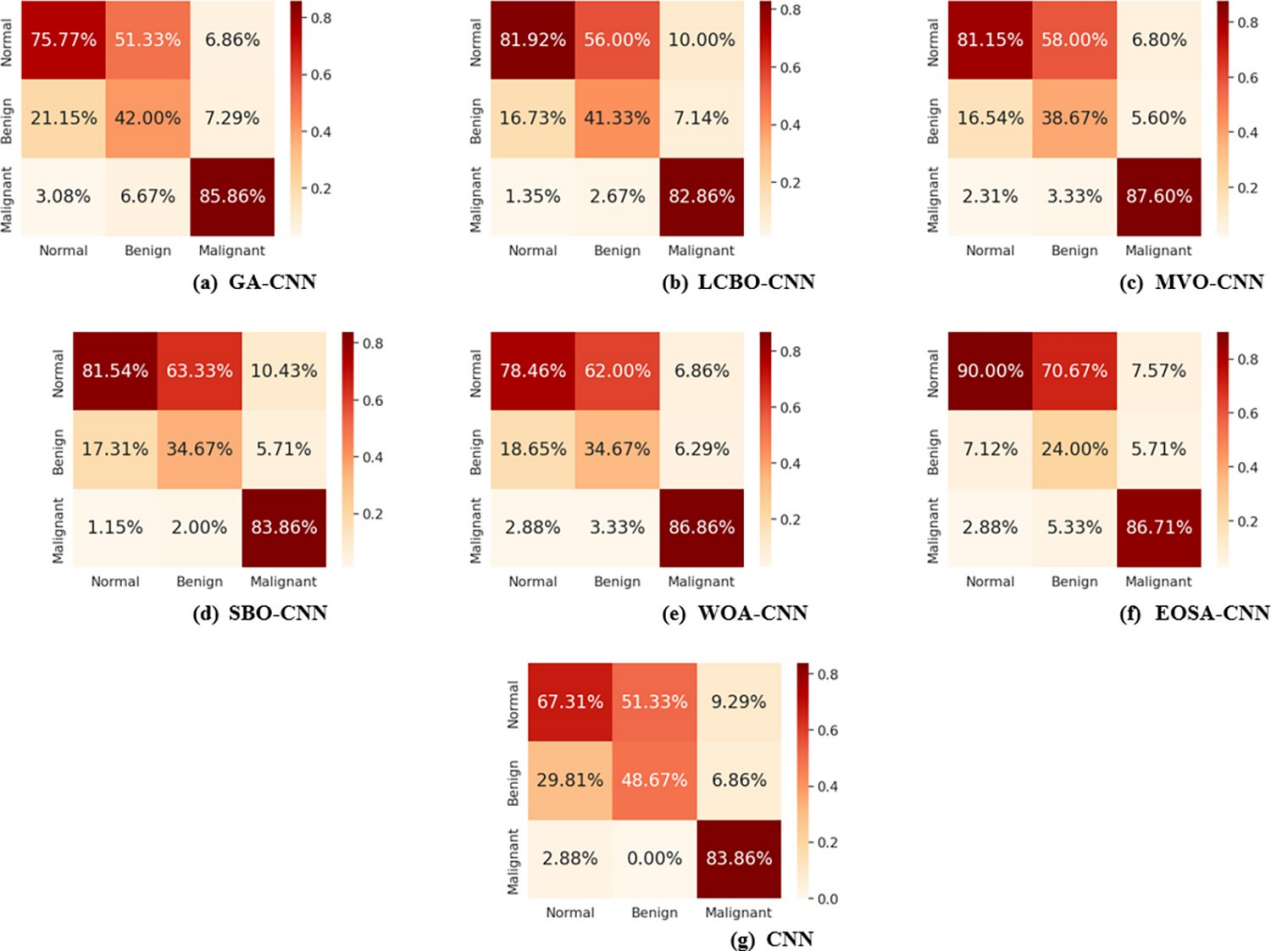
Overlapped confusion matrix for all hybrid algorithms with CNN. (a) GA-CNN, (b) LCBO-CNN, (c) MVO-CNN (d) SBO-CNN, (e) WOA-CNN, (f) EOSA-CNN, and (g) CNN.

Fig 16 shows the confusion matrix plot for all hybrid algorithms with respect to all the class labels observed in the dataset. The classification accuracy of all classes is indicated for each plot of the confusion matrix to give an accurate report on their performances. Taking the case of EOSA-CNN as an example, we see that 90% of all cases with *normal* labels were correctly identified, and over 86% of cases labelled as malignant were correctly identified by the hybrid algorithm proposed in this study. This is contrary to what is reported for the traditional CNN, where only 67.31% of samples with *normal* labels were correctly identified, while about 83% of those with malignancy were correctly identified. This reinforces the impact of the hybrid algorithm proposed in this study since it improved classification accuracy.

In Table 9, we compare the performance of the proposed EOSA-CNN hybrid algorithm with those reported in similar studies. The classification accuracy obtained in the approach proposed in this study competes with those seen in the works of Chen et al. [61] Sultana et al. [62], Bangare et al. [63] Al-Yasriy et al. [64] Dass and Kumar [65] and Lyu [66]. All the similar methods applied basic and benchmark CNN architectures with known use of any parameter optimization strategy. Although the result obtained by most of the studies are interesting, we note that such models will under-perform when some performance tilting conditions are introduced. These approaches are far below that proposed in this study, which aimed to stabilize and solve classification problems using optimized CNN architectures. As seen in this study, we argue that the hyperparameter optimization applied using metaheuristic algorithms promises a stabilized model that learns the classification problem effectively and can address the underlying condition. Therefore, the approach can eliminate false positive rates (FPR) and false negative rates (FNR), often making a mal-trained model yield pseudo-performance. Furthermore, several studies have confirmed that optimizing the architectural configuration of CNN models has now become the state-of-the-art (SOTA) in yielding the best-performing classification models. Therefore, considering such a SOTA approach, which resulted in an impressive performance, demonstrates that classification problem-solving is reliable.

**Table 9:** Performance comparison of the proposed method and some similar methods of CNN for the classification of lung cancer.

Author and Reference	Method	Dataset	Performance
Chen et al., [61]	CNN+ Natural language processing (NLP)	IQ-OTH/NCCD dataset	Accuracy of 88.0%
Sultana et al., [62]	2-D CNN with SVM, ResNet-50, InceptionResNetV2, Inception-V3, and VGG-19	IQ-OTH/NCCD dataset	Accuracy 99.13%
Bangare et al., [63]	CNN model	IQ-OTH/NCCD dataset	Accuracy: 86.42%; Specificity: 86.72%; and Sensitivity: 86.11%
Al-Yasriy et al., [64]	CNN: AlexNet architecture	IQ-OTH/NCCD dataset	Accuracy: 93.548%; Sensitivity: 95.714%; Specificity: 95%
Dass and Kumar [65]	Deep ensemble Convolution neural network (DECNN)	IQ-OTH/NCCD dataset	Accuracy of 99.80%.
Lyu [66]	CNN models: AlexNet, VGG, DCNN and DenseNet	IQ-OTH/NCCD dataset	Accuracy: 97.48% and AUC: 0.99019
**This study**	**EOSA-CNN and selected preprocessing methods**	**IQ-OTH/NCCD lung cancer dataset**	**Accuracy of 93.21%, Sensitivity of 90.71%, Specificity of 1.0, Precision 0f 1.0, F1-score of 92.72% Recall of 90.71%**

In this study, the result of specificity and precision, which are 1.0 for both cases as obtained, confirms that classification accuracy alone is insufficient to demonstrate the methods’ superiority. It can be seen that the proposed method in this study gave a very good performance in its ability to eliminate the presence of false positives and ensured that our model correctly classified negative cases as negative and positive cases as positive. Also, the value of 1.0 for specificity reported for the method proposed in this study showed that the total number of negative cases (normal and benign) in our datasets discovered to be truly negative was very accurate. That means all negative cases were truly confirmed negative by our method. This is very important to rule out the possibility of false negative and false positive results. Yielding a zero level for false positive and false negative rates, as seen by our proposed method, showed that the EOSA-CNN hybrid algorithm is good for classification accuracy and obtains results. This will boost confidence in the resulting output of the proposed algorithm when deployed for use. Therefore, this study has demonstrated the importance of using the hybrid metaheuristic algorithm and CNN models to solve the difficult problem of selecting the best combination of weights and biases required for training a CNN model. Moreover, the approach demonstrates that combining the methods can improve classification accuracy and the general performance of classifying lung cancer in CT images.

## 6. Study limitations

The study has a few limitations, including insufficient data sample size and the lack of consideration for possible imbalanced data and time complexity due to limited resources. We suggest that future work should address these limitations by using techniques such as random under and over-sampling or cluster-based over-sampling and incorporating larger sample sizes to improve model performance. Despite these limitations, the proposed EOSA-CNN model outperformed other hybrid algorithms and traditional CNNs on all seven metrics evaluated, which is significant compared to previous studies. Further research is necessary to evaluate the EOSA model’s performance on other medical problems.

## 7. Conclusion

This study presents a novel hybrid algorithm to improve the accuracy of lung cancer classification using a CNN model. The EOSA algorithm was used to optimize the solution vector of the CNN architecture, which was trained on distinct 2D samples categorized based on their abnormalities. The resulting model performed well on new datasets, indicating its generalization ability. The EOSA-CNN algorithm outperformed traditional CNN and other metaheuristic-based hybrid algorithms, as demonstrated by accuracy, kappa, precision, recall, F1 score, specificity, and sensitivity metrics. The contribution of this study is the successful use of the EOSA algorithm, a virus-based optimization technique, to improve the solution vector of the proposed CNN architecture. Future work includes optimizing the hyperparameters of the CNN model, investigating the possibility of using the hybrid approach to auto-design the CNN architecture and comparing the proposed CNN architecture against benchmarked models for further evaluation. Overall, this study provides a promising classification model for identifying malignant and benign lung cancer cases from digital images, with potential applications in early detection and improved decision-making for patient treatment.
